# Transcriptomic Effects of the Cell Cycle Regulator LGO in *Arabidopsis* Sepals

**DOI:** 10.3389/fpls.2016.01744

**Published:** 2016-11-22

**Authors:** Erich M. Schwarz, Adrienne H. K. Roeder

**Affiliations:** ^1^Department of Molecular Biology and Genetics, Cornell University, IthacaNY, USA; ^2^Weill Institute for Cell and Molecular Biology and Section of Plant Biology, School of Integrative Plant Sciences, Cornell University, IthacaNY, USA

**Keywords:** *loss of giant cells from organs (lgo)*, *siamese related1 (smr1)*, endoreduplication, endoreplication, *Arabidopsis thaliana*, RNA-seq, sepal, transcriptome

## Abstract

Endoreduplication is a specialized cell cycle in which DNA replication occurs, but mitosis is skipped creating enlarged polyploid cells. Endoreduplication is associated with the differentiation of many specialized cell types. In the *Arabidopsis thaliana* sepal epidermis endoreduplicated giant cells form interspersed between smaller cells. Both the transcription factor *Arabidopsis thaliana* MERISTEM LAYER1 (ATML1) and the plant-specific cyclin dependent kinase inhibitor LOSS OF GIANT CELLS FROM ORGANS (LGO)/SIAMESE RELATED1 (SMR1) are required for the formation of giant cells. Overexpression of LGO is sufficient to produce sepals covered in highly endoreduplicated giant cells. Here we ask whether overexpression of LGO changes the transcriptome of these mature sepals. We show that overexpression of LGO in the epidermis (*LGOoe*) drives giant cell formation even in *atml1* mutant sepals. Using RNA-seq we show that *LGOoe* has significant effects on the mature sepal transcriptome that are primarily ATML1-independent changes of gene activity. Genes activated by *LGOoe*, directly or indirectly, predominantly encode proteins involved in defense responses, including responses to wounding, insects (a predator of *Arabidopsis*), and fungus. They also encode components of the glucosinolate biosynthesis pathway, a key biochemical pathway in defense against herbivores. *LGOoe*-activated genes include previously known marker genes of systemic acquired resistance such as *PR1* through *PR5*. The defensive functions promoted by *LGOoe* in sepals overlap with functions recently shown to be transcriptionally activated by hyperimmune *cpr5* mutants in a LGO-dependent manner. Our findings show that the cell cycle regulator LGO can directly or indirectly drive specific states of gene expression; in particular, they are consistent with recent findings showing LGO to be necessary for transcriptional activation of many defense genes in *Arabidopsis*.

## Introduction

During development, particular cell types often have specialized cell cycles. In plants, many differentiating cell types undergo endoreduplication, a cell cycle in which cells grow and replicate their DNA but do not undergo mitosis or cytokinesis (Lee et al., 2009; Breuer et al., 2010). Likewise, in animals, endoreduplication is linked with differentiation of several key cell types, such as megakaryocytes that generate platelets in mammalian blood and salivary gland cells in *Drosophila* that contain polytene chromosomes (Ullah et al., 2009). Endoreduplication allows cells to become enlarged, and the endopolyploidy level (i.e., DNA content) is directly proportional to cell size (Melaragno et al., 1993; Roeder et al., 2010).

**FIGURE 1:**
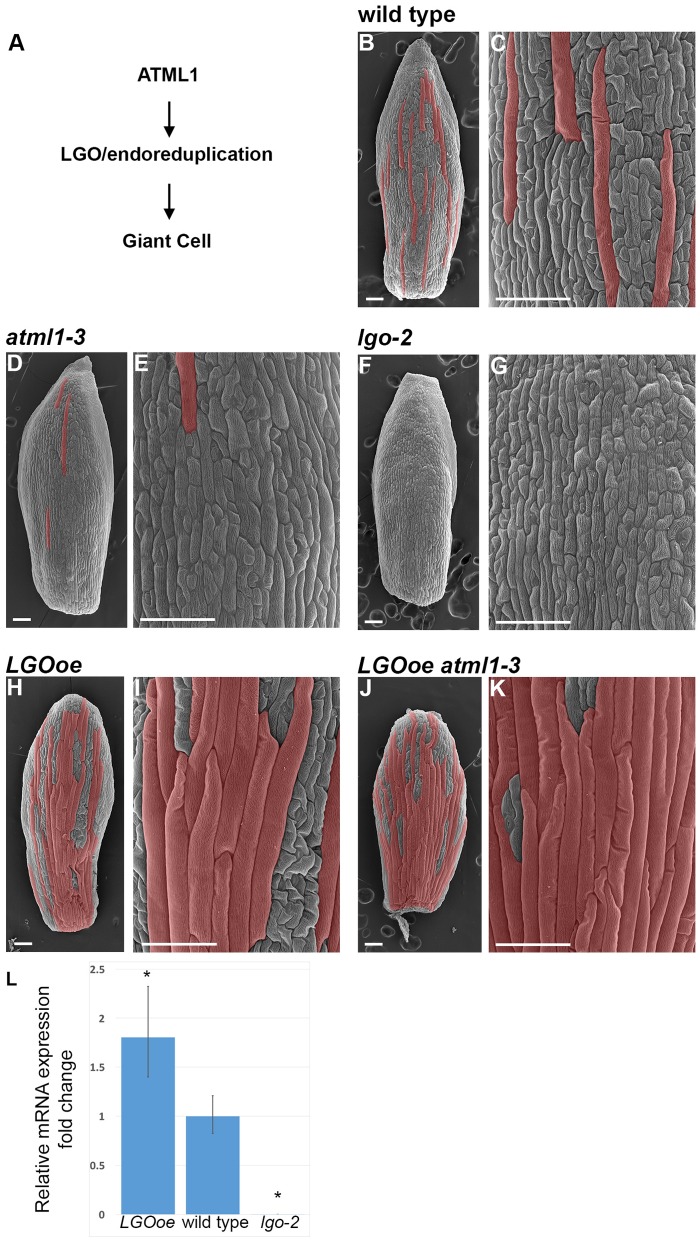
**Cell size patterning phenotypes.** **(A)** A genetic diagram depicting our current understanding of the pathway leading to giant cell formation. The HD-ZIP class IV transcription factor ATML1 directly or indirectly activates endoreduplication through the CKI LGO. Endoreduplication causes the enlargement and differentiation of the cell. It is not yet known to what extent LGO, endoreduplication, or both regulate gene expression in the sepal. **(B,C)** Scanning electron micrograph (SEM) of a wild-type sepal at stage 12. Giant cells, as assessed by their size and outward bulging from the surface of the sepal, have been false-colored red in Photoshop. **(C)** Is a magnified view of the cells in **(B)**. **(D,E)** SEM of *atml1-3* stage 12 mutant sepal **(D)** and magnified view of the cells **(E)**. Giant cells are strongly reduced in this allele, although the phenotype is not as strong as *lgo-2*. **(F,G)** SEM of *lgo-2* mutant sepal **(F)** at stage 12 with magnified view of the cells **(G)**. Note the absence of giant cells. **(H,I)** SEM of a stage 12 sepal in which *LGO* is overexpressed throughout the epidermis under control of the *ATML1* promoter (*ATML1p::LGO*); this genotype is referred to throughout the text as *LGOoe*. Many highly enlarged cells are formed that appear similar to giant cells in phenotype. **(J,K)** SEM of a sepal in which *LGO* is overexpressed in the epidermis of an *atml1-3* mutant (*LGOoe atml1-3*). Many highly enlarged cells form, suggesting that LGO acts downstream of ATML1 in the formation of giant cells. This genotype allowed us to determine what genes LGO could regulate independently of ATML1 activity. Sepals in all of the images were taken from replicate 1 plants. **(L)** qPCR measurement of the 1.8-fold increase of *LGO* expression in inflorescences from *LGOoe* plants relative to Col_WT inflorescences. With these primers which flank the t-DNA insertion site, no *lgo-2* transcript is detected. ^∗^ indicates *p*-value < 0.05 when compared to wild type. Error bars represent the standard deviation.

The *Arabidopsis* sepal epidermis is a new model system in which to investigate the role of endoreduplication in the formation of specialized giant cells. The sepal is the outermost green floral organ, which encloses and protects the developing reproductive organs. The cells in the outer/abaxial epidermis of *Arabidopsis* sepals are diverse in size, ranging from giant cells stretching to an average of 360 μm, to the smallest cells reaching only about 10 μm (**Figures 1A–C**) (Roeder et al., 2010). Giant cells are also found on the abaxial epidermis of leaves (Melaragno et al., 1993; Roeder et al., 2010, 2012). A key function of giant cells is precise control of the curvature of sepals, which is necessary for sepals to form a closed shell protecting immature flowers (Roeder et al., 2010, 2012). In the sepal epidermis, cell types are correlated with variations in cell cycles. Giant cells generally undergo three rounds of endoreduplication to become endopolyploid 16C cells, whereas small cells undergo mitotic divisions and remain generally 2C or 4C (Roeder et al., 2010). Two enhancer trap markers drive cell type-specific expression within the sepal, one in giant cells and the other in small cells; these enhancers demonstrate that giant cells and small cells can have distinct patterns of gene expression, as well as distinct cell sizes and DNA contents (Roeder et al., 2012). Moreover, study of these enhancers in mutant backgrounds has shown that the balance between giant and small cells in sepals depends both on the transcription factor gene *ATML1* and on the cell cycle regulator gene *LGO*.

Mutations in *atml1* result in the reduction or absence of giant cells in sepals, and the corresponding loss of 16C cells in the epidermis (**Figures 1D,E**) (Roeder et al., 2012). *ATML1* encodes a HD-ZIP IV transcription factor and is important for establishing epidermal identity together with its paralog, PROTODERMAL FACTOR2 (PDF2) (Abe et al., 2003; Nakamura et al., 2006). The epidermis is absent in *atml1 pdf2* double mutants, exposing the mesophyll cells, whereas *atml1* single mutants have an intact epidermis, but lack giant cells. Overexpression of ATML1 or the related HD-ZIP protein HDG2 in internal cell layers of the cotyledon is sufficient to induce the ectopic formation of epidermal cell types including giant cells and stomata (Peterson et al., 2013; Takada et al., 2013). ATML1 promotes expression of the giant cell molecular marker: in *atml1* sepals, its expression significantly diminishes (Roeder et al., 2012). Conversely, ATML1 has little effect on expression of the small cell molecular marker, which remains largely unchanged in *atml1* sepals.

Similarly to *atml1*, *lgo* mutants fail to form giant cells because all the epidermal cells in sepals and leaves divide instead of endoreduplicating, creating numerous small cells in the place of giant cells (**Figures 1F,G**) (Roeder et al., 2010). Ploidy measurements confirm that 16C giant cells are absent in *lgo* mutants. Conversely, overexpression of *LGO* throughout the epidermis (herein referred to as *LGOoe*) causes the abaxial sepal epidermis to be nearly covered with highly endoreduplicated giant cells (**Figures 1H,I**) (Roeder et al., 2010), because epidermal cells that would normally divide to make small cells instead endoreduplicate to make giant cells. *LGO*, also known as *SIAMESE RELATED1* (*SMR1)*, encodes a cyclin dependent kinase inhibitor (CKI) in the plant-specific family of SIAMESE RELATED (SMR) homologs, whose founding member is *SIAMESE* (*SIM*). SMRs bind to and inhibit cyclins and CDKs, promoting endoreduplication (Churchman et al., 2006; Peres et al., 2007; Roeder et al., 2010; Kumar et al., 2015). LGO has little effect on expression of the giant cell molecular marker, which is still expressed in *lgo* mutant sepals (that lack overt giant cells) and whose expression does not increase in *LGOoe* sepals (that are dominated by excess giant cells). Conversely, LGO has strong effects on expression of the small cell molecular marker: in *lgo* sepals, its expression spreads to most cells in the sepal; in *LGOoe* sepals, its expression shrinks to fewer cells.

There are at least two ways that CKIs such as LGO could affect gene expression. First, CKIs can affect gene expression through their regulation of the cell cycle. CKIs inhibit the activity of cyclin dependent kinases (CDKs) (Kumar et al., 2015). The LGO paralog SIM has been shown to interact with both CYCLIN Ds and CYCLIN DEPENDENT KINASE A;1 (CDKA;1) which regulate the G1 to S transition in the cell cycle (Churchman et al., 2006; Kumar et al., 2015). CYCLIND/CDKA;1 complexes phosphorylate RETINOBLASTOMA RELATED1 (RBR1), releasing E2F transcription factors to activate cell cycle gene transcription (Desvoyes et al., 2006, 2014). RBR1 can bind to and regulate the activity of other transcription factors including the key developmental regulators FAMA and SCARECROW (Cruz-Ramírez et al., 2012; Matos et al., 2014). In addition, CDKs can phosphorylate transcription factors to regulate their activity (Reindl et al., 1997; Yang et al., 2015). Second, in animals it has been found that CKIs can directly control gene expression (Lim and Kaldis, 2013) by binding transcription factors and altering their activity by either activating or inhibiting target genes (Li et al., 2012; Pippa et al., 2012; Lawrence et al., 2014; Hardwick et al., 2015). Similar mechanisms are likely in plants. In a large-scale survey of *Arabidopsis* cell cycle protein complexes, four p27^Kip1^-related CKIs (KRP2, KRP3, KRP4, and KRP5) were found to bind four different transcription factors; LGO was found to bind the transcription factor bZIP69 (Van Leene et al., 2010). ChIP-seq has demonstrated that one of these *Arabidopsis* CKIs, KRP5, binds 264 genes that are enriched 23-fold for functions in cell wall organization, and that may be transcriptionally activated by KRP5 (Jegu et al., 2013). Likewise, a CKI in tomato forms part of a protein complex with the bZIP transcription factor SPGB (Pnueli et al., 2001; Churchman et al., 2006). Meanwhile, genetic analysis of the CKI SIAMESE (SIM) in *Arabidopsis* has demonstrated that it is required for cell fate: some *siamese* mutant trichomes (leaf hair cells) that fail to undergo endoreduplication lose their trichome identity, and revert to pavement cells (Bramsiepe et al., 2010).

Here, we use RNA-seq to determine to what extent LGO controls sepal gene expression directly or indirectly in mature sepals, independently of ATML1.

## Results

### Overexpression of LGO Promotes Giant Cell Formation Even in the Absence of ATML1

To determine to what degree LGO affects gene expression in mature sepals independently of ATML1, we first generated plants in which *LGO* was ectopically expressed under the epidermal-specific ATML1 promoter (referred to as *LGOoe*) (Sessions et al., 1999; Roeder et al., 2010, 2012) in the wild-type Columbia (Col_WT) background. *LGO* expression increased by 1.8-fold in inflorescences of the *LGOoe* plants (**Figure 1L**). We crossed the *LGOoe* plants to *atml1-3* mutants and isolated plants homozygous for both *LGOoe* and *atml1-3* (**Figures 1J,K**). We have previously shown that overexpressing *KRP1* in a *atml1-3* mutant background is sufficient to induce large, highly endoreduplicated cells (Roeder et al., 2012). Similarly, overexpressing *LGO* throughout the epidermis in either wild-type (**Figures 1H,I**) or an *atml1-3* mutant background (**Figures 1J,K**) produced plants with highly enlarged cells, morphologically indistinguishable from giant cells, that largely covered the outer sepal epidermis. The existence of giant cells in *LGOoe atml1-3* sepals (**Figure 1A**) confirmed previous work indicating that *LGO* exerts its effects on sepal endoreduplication and differentiation downstream of *ATML1* and other epidermal specification genes (Roeder et al., 2012).

### Transcriptomic Analysis Identifies Genes Regulated by LGO Independently of ATML1

We then used RNA-seq to observe and compare the transcriptomes of wild-type Columbia sepals (Col_WT; **Figures 1B,C**), *atml1-3* sepals (**Figures 1D,E**), *lgo-2* sepals (**Figures 1F,G**), *LGOoe* sepals (**Figures 1H,I**), and *LGOoe atml1-3* sepals (**Figures 1J,K**). Three batches of these five genotypes were grown and harvested for RNA-seq, providing three biological replicates per genotype. For each replicate, we performed RNA-seq on 250 dissected whole stage 12 sepals. At stage 12, cells in the sepal have fully differentiated and cell division and endoreduplication has been completed (Roeder et al., 2012; Hervieux et al., 2016; Hong et al., 2016). This strategy allowed us to assess sepal transcriptomes in their final, differentiated state: expressing terminal genes that might be driven by LGO in mature sepals, rather than others (e.g., cell-cycle genes, or developmental regulatory genes) that might be driven by LGO in earlier stages of sepal development.

**FIGURE 2:**
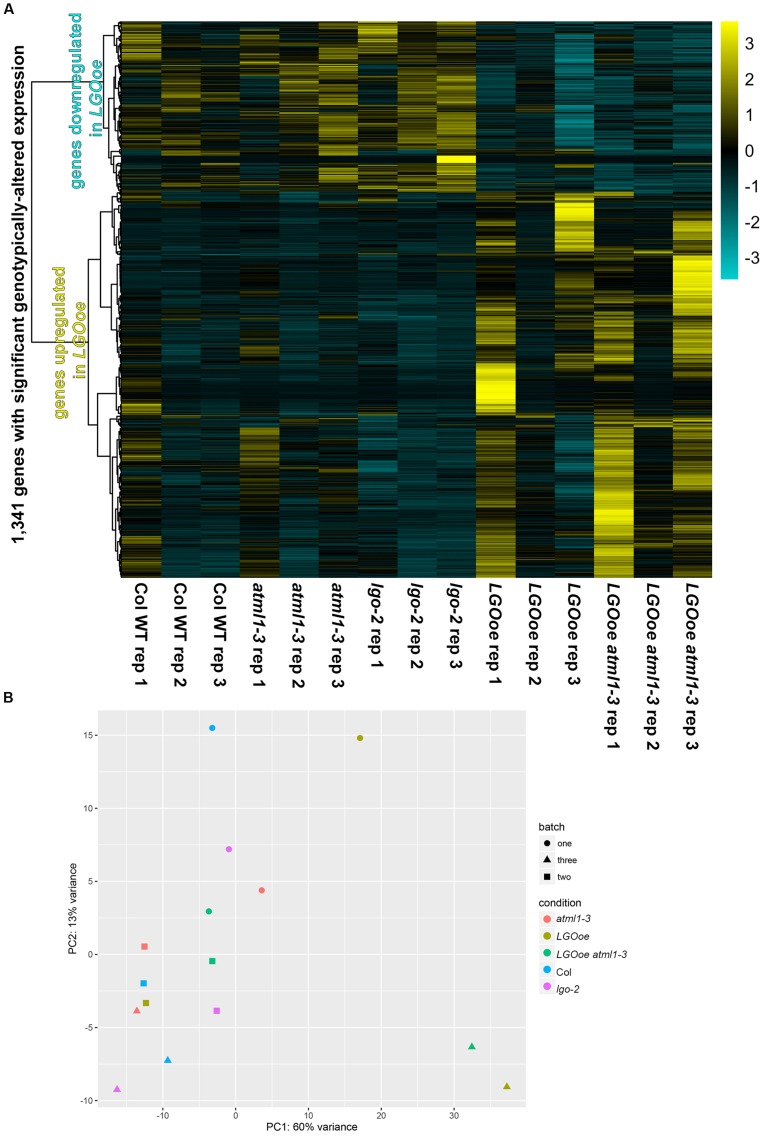
**Gene expression in sepals.** **(A)** An overview of relative gene activity for the 1,341 genes with significant differences in expression between genotypes. For each gene, expression levels were centered and scaled, so that the relative expression levels between conditions for the gene (rather than absolute changes of its expression levels) are shown. On the left, a dendrogram showing the clustering of these relative expression patterns is given. Relative expression is graded from maximum (yellow) to minimum (dark turquoise). The most obvious distinction in gene expression is between *LGOoe* and non-*LGOoe* genotypes; other changes in genotype have much weaker effects. Meanwhile, there are also visible differences between batches, with replicate 1 being obviously different from replicates 2 and 3. This probably reflects the effects of subtle environmental variations upon gene activity in sepals. **(B)** A principal component analysis (PCA) of each genotype (represented by color) in each batch (represented by shape) showing the differences in gene expression between genotypes and replicates. Note that samples in which LGO is overexpressed tend to fall toward the right of their respective batch on PC1.

We used DESeq2 (Love et al., 2014) to identify 1,341 genes with significant differences in expression between genotypes, taking into account the three biological replicate batches of the samples (**Figure 2**; **Supplementary File S01**). Between genotypes, the strongest differences in gene expression were caused by *LGO* overexpression (**Figure 2**; **Table 1**; **Supplementary File S02**). More genes were significantly upregulated by *LGO* overexpression than downregulated (**Figure 2**; **Table 1**; **Supplementary File S02**). Expression trends were similar in *LGO* overexpression and *LGO* overexpression in the *atml1* mutant background (*LGOoe* versus *LGOoe atml1-3*), suggesting that *LGO* regulates gene expression largely independently of *ATML1* (**Figure 2**; see further analysis below). In addition, there was a substantial variability in expression of these significantly differentially expressed genes between replicates; however, all of these genes had significant *p*-values for differential expression based on genotypes controlling for variation between replicate batches (**Figure 2**; see further analysis below).

**Table 1:** Genes with conditionally changed expression.

Conditional change	Genes significantly up	Genes significantly down
Comparisons between genotypes:		
Col_WT vs. *atml1-3*	1	1
Col_WT vs. *lgo-2*	5	10
*atml1-3* vs. *lgo-2*	1	13
*LGOoe* vs. Col_WT	132	24
*LGOoe* vs. *atml1-3*	305	57
*LGOoe* vs. *lgo-2*	392	132
*LGOoe atml1-3* vs. Col_WT	228	82
*LGOoe atml1-3* vs. *atml1-3*	203	89
*LGOoe atml1-3* vs. *lgo-2*	584	322
*LGOoe* vs. *LGOoe atml1-3*	25	5
Comparisons between batches:		
Batch two vs. batch one	1,275	1,527
Batch three vs. batch one	1,229	1,290
Batch three vs. batch two	836	415


*For each change between two conditions (genotype or batch, listed as “B vs. A”) and each direction of change (increased or decreased gene expression in B, with respect to A), the number of genes changing expression with statistical significance (as evaluated by DESeq2; see Materials and Methods) is given.*

In contrast, only two genes were significantly differentially expressed between Col_WT and *atml1-3* mutants (**Table 1**; **Supplementary File S02**). Likewise only 15 genes were significantly differentially expressed between Col_WT and *lgo-2* mutants (**Table 1**; **Supplementary File S02**). Given that there are only about 20 giant cells per sepal out of about 1,600 outer epidermal cells and many more cells in the other cell layers, it is not surprising that transcriptomic differences between Col_WT, *atml1-3* and *lgo-2* sepals would be small (**Figure 1**).

To double-check our analysis and ensure its accuracy, given that in some comparisons we found very few significantly differentially expressed genes, we used a second statistical analysis software package (edgeR) to reanalyze the RNA-seq data. edgeR identified exactly the same two genes and only those two genes as differentially expressed between Col_WT and *atml1-3* mutant sepals. Furthermore, for each comparison, edgeR identified fewer genes as significantly differentially expressed between the genotypes. There was nearly complete overlap between the genes identified as differentially expressed by edgeR and the larger set of genes identified by DESeq2 (**Supplementary File S03**). This reanalysis validated the low number of differentially expressed genes identified by DESeq2; therefore, we used the DESeq2-derived genes for further analysis.

In the analyses below, we have focused on differential gene expression caused by *LGO* overexpression. In many cases, this led us to compare a gain-of-function mutant genotype (e.g., *LGOoe*) to a loss-of-function mutant genotype (e.g., *lgo-2*), rather than to Col_WT alone.

**FIGURE 3:**
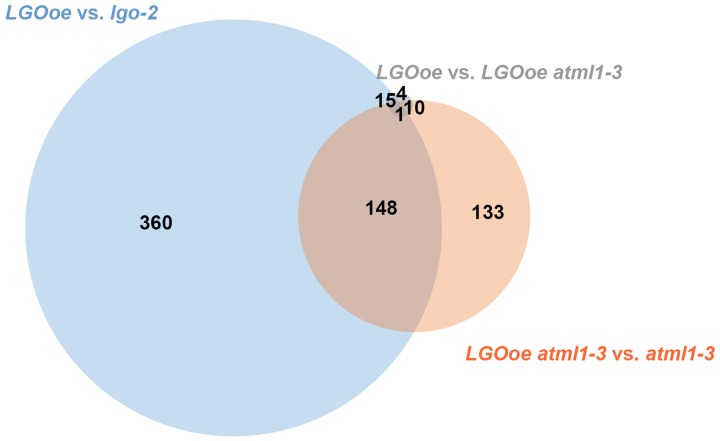
***LGOoe* differentially expressed genes.** Venn diagram showing the number of differentially expressed genes shared between three different genotypic comparisons: *LGOoe* versus *lgo-2* (blue), *LGOoe atml1-3* versus *atml1-3* (orange), and *LGOoe* versus *LGOoe atml1-3* (gray). Note that only 30 genes are differentially expressed between *LGOoe* versus *LGOoe atml1-3*, suggesting that very few of the genes regulated by *LGOoe* require ATML1 activity.

We next asked to what extent LGO affects transcription in the mature sepal independently of ATML1. We found that 292 genes were differentially expressed when *LGO* was overexpressed in the *atml1-3* mutant when compared to the *atml1-3* mutant alone (*LGOoe atml1-3* versus *atml1-3*; **Table 1**; **Supplementary File S02**), indicating that the change of expression of these genes was independent of *ATML1*. 149 of these genes were also significantly differentially regulated when *LGOoe* was compared with *lgo-2*, confirming that these genes are regulated by LGO (**Figure 3**). Thus, many genes are directly or indirectly regulated by *LGO* independent of *ATML1*. Conversely, only 30 genes were significantly differentially regulated between *LGOoe* and *LGOoe* in the *atml1-3* mutant (*LGOoe* versus *LGOoe atml1-3*), suggesting that very few genes that are regulated by *LGO* depend on ATML1 activity (**Table 1**; **Figure 3**). Therefore, a large majority of genes whose transcription was either up- or down-regulated by *LGOoe* did not depend on ATML1 activity, which is consistent with the role of LGO downstream of ATML1 in the giant cell formation pathway (**Figure 1A**).

As a positive control, we examined expression of AT5G17700, a MATE transporter gene that we have previously shown contains an enhancer driving giant cell-specific expression in sepals (**Table 2**) (Roeder et al., 2012). As expected, AT5G17700 expression was significantly lower in *lgo-2* mutant sepals than wild type (2.4-fold higher expression in Col_WT versus *lgo-2*, padj = 2.3 ⋅ 10^-2^; **Supplementary File S01**), consistent with the diminished levels of giant cell enhancer expression observed in the small cells of *lgo-2* mutant sepals (Roeder et al., 2012). A slight downregulation of AT5G17700 in *atml1* mutants (1.9-fold higher expression in Col_WT versus *atml1*) did not reach statistical significance (*p* = 1.2 ⋅ 10^-3^, but padj = 1 after multiple testing correction). However, it did match previous observations of decreased giant enhancer expression in *atml1* mutant sepals. AT5G17700 expression in either *LGOoe* or *LGOoe atml1* genotypes was somewhat lower than its expression in Col_WT (*LGOoe*, 1.5-fold lower, *p* = 4.4 ⋅ 10^-2^, padj = 0.37; *LGOoe atml1*, 1.3-fold lower, *p* = 0.21, padj = 0.87); this fit our previous finding that endoreduplication alone does not activate expression of the giant cell enhancer (Roeder et al., 2012).

**Table 2:** Expression of giant cell enhancer gene AT5G17700.

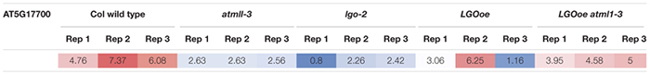

*Heat map (red high, blue low) showing the expression values (TPM) for AT5G17700, the gene downstream of a giant cell-specific enhancer (Roeder et al., 2012). AT5G17700 expression is downregulated in *lgo-2*, but not upregulated in *LGOoe*. This expression pattern matches that of the giant cell enhancer in mutant backgrounds, validating the RNA-seq data.*

We noticed considerable variability in expression of genes regulated by *LGOoe* between replicates (**Figure 2**) and many of the genes differentially regulated in *LGOoe* sepals were associated with response to the biotic environment (see below), so we examined the differences between biological replicates (batches). When we examined the expression of all genes, our biological replicates were highly reproducible with high correlation (*R*^2^) in gene expression between replicates (**Supplementary File S04**). However, we found 3,954 genes with significant changes of expression between batches but not genotypes, 598 genes with significant changes between genotypes but not batches, and 743 genes with significant changes between both batches and genotypes (**Supplementary File S01**); importantly, DESeq2 enabled statistical analyses in which changes in one condition (e.g., genotype) were controlled for simultaneous changes in a different condition (e.g., batch). Despite large changes in gene activity between the three batches, we observed no obvious differences in their health or growth conditions; nor did we observe any obvious sources of stress, such as wounding. Sepals are protective organs, and plants are thought to use transcriptional responses to cope with the challenges of their sessile lifestyle; thus, the changes in gene activity between batches may have been authentic *in vivo* responses to subtle environmental variations. In fact, these environmental variations were advantageous, allowing us to identify genes whose expression responded both to *LGOoe* and the environment (e.g., PR1 through PR5; see below).

### Defense Responses are Upregulated by LGO Overexpression

To identify biological and molecular functions driven by LGO overexpression in sepal transcriptomes, we used FUNC (Prüfer et al., 2007) to identify GO terms that were statistically overrepresented among genes whose expression significantly changed between either genotypes or batches. To filter out environmental effects, we sorted the resulting GO terms into three groups (**Supplementary File S05**): those for which the most significant effect (as measured by *p*-value) came from a changed genotype rather than a changed batch (used for further analysis); those for which the most significant effect came from a changed batch comparison, but that did have significant *p*-values for changed genotypes as well; and those GO terms that only had significant *p*-values for changed batches. Because the first group of GO terms described functions more strongly affected by genotypic than environmental changes, we considered it most likely to describe the effects of LGO on sepal transcriptomes. Within this group, we identified several distinct subsets of GO terms that were associated with different genotypic changes and varying degrees of dependence on ATML1 (**Tables 3**–**5**; **Supplementary Files S06**–**S10**). In our sample harvesting technique, sepals were snap-frozen immediately upon dissection; we therefore consider it unlikely that the biological effects described below were induced by our experimental procedures.

**Table 3:** Functions upregulated in sepals by *LGOoe* despite the absence of ATML1.

GO term	*p*-value	Gene count
Response to wounding [GO:0009611]	1.29926e-18	43
Response to chitin [GO:0010200]	5.23826e-11	25
Defense response by callose deposition in cell wall [GO:0052544]	4.10392e-09	8
Induced systemic resistance [GO:0009682]	4.83273e-09	10
Defense response [GO:0006952]	1.01999e-08	68
Glucosinolate biosynthetic process [GO:0019761]	7.67952e-08	10
Response to insect [GO:0009625]	8.8687e-07	10
Defense response to fungus, incompatible interaction [GO:0009817]	1.90923e-06	9
Regulation of systemic acquired resistance [GO:0010112]	2.08231e-05	4
Indoleacetic acid biosynthetic process [GO:0009684]	7.56308e-05	5
Oxygen binding [GO:0019825]	8.54446e-05	28
Heme binding [GO:0020037]	9.22733e-05	35
Cellular heat acclimation [GO:0070370]	0.000112295	4
Response to herbivore [GO:0080027]	0.000112295	3
Defense response to fungus [GO:0050832]	0.000183117	38
Tryptophan catabolic process [GO:0006569]	0.00021621	2
Oxidoreductase activity, acting on paired donors, with incorporation or reduction of molecular oxygen, NAD(P)H as one donor, and incorporation of one atom of oxygen [GO:0016709]	0.000228724	19
Response to ethylene [GO:0009723]	0.000278924	19
Allene-oxide cyclase activity [GO:0046423]	0.000481594	2
Sucrose transmembrane transporter activity [GO:0008515]	0.000535353	3
Respiratory burst [GO:0045730]	0.000540456	1
Jasmonic acid mediated signaling pathway [GO:0009867]	0.000553934	7
Sucrose transport [GO:0015770]	0.000604359	4
Palmitoyl-(protein) hydrolase activity [GO:0008474]	0.000777282	2
Thiosulfate sulfurtransferase activity [GO:0004792]	0.000797888	1
Response to desiccation [GO:0009269]	0.000848643	4
Plant-type vacuole [GO:0000325]	0.00107121	5
Hydrogen sulfide biosynthetic process [GO:0070814]	0.00113603	3
Regulation of response to water deprivation [GO:2000070]	0.00143782	2
Adenylylsulfate kinase activity [GO:0004020]	0.00165573	2
Phosphatidylethanolamine binding [GO:0008429]	0.00165573	2
Regulation of glucosinolate biosynthetic process [GO:0010439]	0.00166834	3
Indole glucosinolate metabolic process [GO:0042343]	0.00192757	3
ER body [GO:0010168]	0.00217588	2
L-aspartate:2-oxoglutarate aminotransferase activity [GO:0004069]	0.00280486	2
Response to cadmium ion [GO:0046686]	0.00285117	12
Iron ion binding [GO:0005506]	0.00513345	31


*GO term describes functions significantly overrepresented among genes that were more strongly expressed in *LGOoe atml1-3* sepals than in *atml1-3* sepals. *p*-value is given for the most significant genotypic change associated with each GO term, which sometimes involved genotypic changes other than *LGOoe atml1-3* versus *atml1-3*. Gene count is given for the number of genes that are associated with the GO term, and that also significantly change expression in a GO term-associated genotypic or batch change; in other words, for genes that are responsible for a GO term being associated with a given change of gene activity. Full details of this GO term subset are given in **Supplementary File S06**.*

**Table 4:** Functions expressed in *LGOoe* sepals in an *ATML1*-dependent manner.

GO term	*p*-value	Gene count
Response to heat [GO:0009408]	1.30164e-23	25
Response to hydrogen peroxide [GO:0042542]	9.83963e-15	13
Response to high light intensity [GO:0009644]	3.19185e-14	13
Plasmodesma [GO:0009506]	7.93474e-07	19
Protein unfolding [GO:0043335]	0.00141278	1


*GO term describes functions significantly overrepresented among genes that were more strongly expressed in *LGOoe* sepals than in *LGOoe atml1-3* sepals. *p*-value is given for the most significant genotypic change associated with each GO term, which sometimes involved genotypic changes other than *LGOoe* versus *LGOoe atml1-3*. Gene count is given for the number of genes that are associated with the GO term, and that also significantly change expression in a GO term-associated genotypic or batch change; in other words, for genes that are responsible for a GO term being associated with a given change of gene activity. Full details of this GO term subset are given in **Supplementary File S07**.*

**Table 5:** Functions more expressed in *LGOoe* sepals than in either *lgo-2* or *atml1-3* sepals.

GO term	*p*-value	Gene count
Systemic acquired resistance [GO:0009627]	1.03862e-10	14
Response to virus [GO:0009615]	1.66036e-07	9
Response to fungus [GO:0009620]	2.53993e-05	15
Response to oxidative stress [GO:0006979]	6.63141e-05	36
Camalexin binding [GO:2001147]	0.000173634	2
Quercitrin binding [GO:2001227]	0.000173634	2
Chitin binding [GO:0008061]	0.00020204	4
Indole glucosinolate biosynthetic process [GO:0009759]	0.000217908	2
Cellular response to hypoxia [GO:0071456]	0.000461928	5
Positive regulation of cell death [GO:0010942]	0.00310496	2


*GO term describes functions significantly overrepresented among genes that were both more strongly expressed in *LGOoe* sepals than in *lgo-2* sepals, and more strongly expressed in *LGOoe* sepals than in *atml-1* sepals, but did not significantly change expression either between *LGOoe* versus *LGOoe atml1-3* or between *LGOoe atml1-3* versus *atml1-3. p*-value is given for the most significant genotypic change associated with each GO term, which sometimes involved genotypic changes other than *LGOoe* versus *lgo-2* or *LGOoe* versus *atml1-3*. Gene count is given for the number of genes that are associated with the GO term, and that also significantly change expression in a GO term-associated genotypic or batch change; in other words, for genes that are responsible for a GO term being associated with a given change of gene activity. Full details of this GO term subset are given in **Supplementary File S08**.*

**FIGURE 4:**
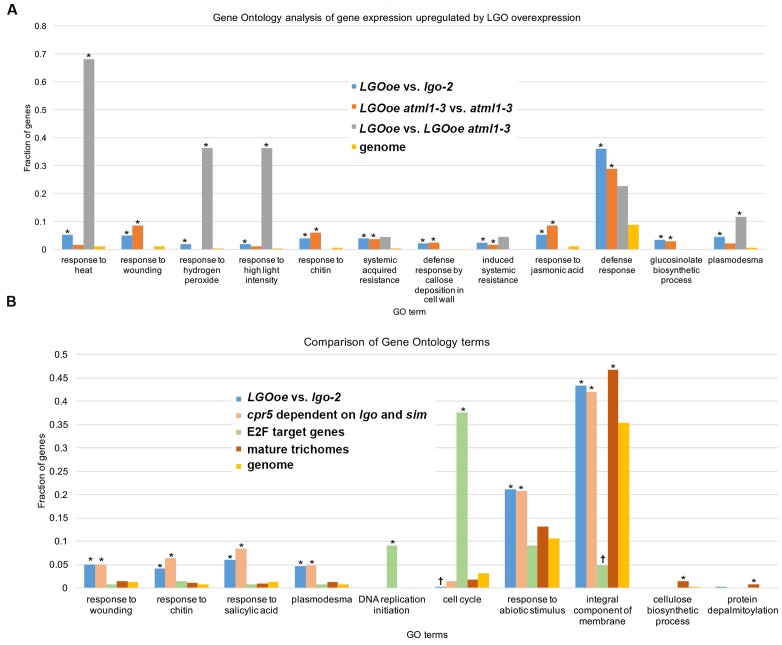
**Defense response is upregulated by *LGO* overexpression in the mature sepal epidermis.** **(A)** Bar graph showing the fraction of genes associated with the GO term in each genotype comparison: *LGOoe* versus *lgo-2* (blue), *LGOoe atml1-3* versus *atml1-3* (orange), and *LGOoe* versus *LGOoe atml1-3* (gray). “Genome” (yellow) reports the frequency of genes associated with that term in the *Arabidopsis* genome, which would be the frequency expected by chance for a randomly selected subset of genes. The fraction of genes was calculated as the number of genes associated with that term divided by the total number of genes in the set that can be associated with any GO term as determined by FUNC. Asterisks indicate significant enrichment (*p*-values ≤ 0.01). Note that many defense response-related GO terms were associated with *LGOoe*, and that only a few terms (such as *response to heat* [GO:0009408] and *response to hydrogen peroxide* [GO:0042542]) required ATML1 activity. **(B)** Bar graph showing the fraction of genes associated with the GO term in each set: *LGOoe* versus *lgo-2* (blue), *cpr5* versus *cpr5 sim lgo* (salmon; genes whose expression in *cpr5* depends on *sim* and *lgo*; Wang et al., 2014), known E2F target genes (green; Vandepoele et al., 2005), mature trichomes (brick red; wild type trichome versus wild type shoot and *gl3-sst sim* mutant; Marks et al., 2009), and genome (yellow). The fraction of genes was calculated as the number of genes associated with that term divided by the total number of genes in the set that can be associated with any GO term as determined by FUNC. Asterisks indicate significant enrichment (*p*-values ≤ 0.01) and daggers (†) indicate significant depletion (*p*-values ≤ 0.01). Note that the frequencies of GO terms associated with *LGOoe* versus *lgo-2* match well with those associated with *cpr5 versus cpr5 sim lgo*, but not with known E2F targets, or with mature trichomes.

Genes with functions in *defense response* [GO:0006952] to many different pathogens including *response to insect* [GO:0009625] and *defense response to fungus* [GO:0050832] were all significantly upregulated in sepals by *LGOoe* versus non-*LGOoe* genotypes (i.e. Col_WT, *atml1-3*, or *lgo-2*; **Tables 3**–**4**; **Figure 4A**; **Supplementary Files S06**, **S07**, **S11**, **S12**). Many GO terms associated with the defense response hormone signaling pathways salicylic acid (*response to salicylic acid* [GO:0009751]), jasmonic acid (*jasmonic acid mediated signaling pathway* [GO:0009867]), and ethylene (*response to ethylene* [GO:0009723]) (Pieterse et al., 2009) have significantly higher representation among the genes upregulated by *LGOoe* versus non-*LGOoe* genotypes than among all protein-coding genes in the *Arabidopsis* genome (**Figure 4**; **Table 3**). Modification of the cell wall at the site of pathogen attack is a common response to infection (Clay et al., 2009; Luna et al., 2011) and *defense response by callose deposition in the cell wall* [GO:0052544] is significantly associated with both *LGOoe* versus *lgo-2* and *LGOoe atml1-3* versus *atml1-3* (**Figure 4A**; **Tables 3**–**4**). PAMP-triggered immunity initiates upon recognition of a common feature of pathogens such as chitin, which is found in the cell wall of fungi and in the exoskeleton of insects (Shibuya and Minami, 2001); *response to chitin* [GO:0010200] is significantly upregulated in both *LGOoe* versus *lgo-2* and *LGOoe atml1-3* versus *atml1-3* (**Figure 4B**; **Table 3**; **Supplementary File S06**) (Shibuya and Minami, 2001). On the other hand, *response to herbivore* [GO:0080027] and *response to wounding* [GO:0009611] that occurs during insect predation induce the *jasmonic acid mediated signaling pathway* [GO:0009867], which are all significantly associated with genes upregulated in *LGOoe atml1-3* versus *atml1-3* (**Figure 4A**; **Table 3**; **Supplementary File S06**) (Leon et al., 2001; Acosta and Farmer, 2010). Plants also recognize pathogen effectors and initiate effector-triggered immunity, which induces a *respiratory burst* [GO:0045730] of reactive oxygen species and *response to hydrogen peroxide* [GO:0042542]; both of these functions are significantly associated with genes upregulated by *LGOoe* versus non-*LGOoe* genotypes (**Figure 4A**; **Tables 3**–**4**; **Supplementary Files S06** and **S07**) (Torres et al., 2006; Suzuki et al., 2011).

This pathway leads to the expression of WRKY transcription factors, which generally modulate transcription of defense response genes, with both positive and negative effects on defense responses (Pandey and Somssich, 2009). *WRKY25* and *WRKY33* were both significantly upregulated in both *LGOoe* versus *lgo-2* and *LGOoe atml1* versus *atml1*, *WRKY51* and *WRKY53* were significantly upregulated in *LGOoe* versus *lgo-2*, and *WRKY26* and *WRKY48* were significantly upregulated in *LOGoe atml1-3* versus *atml1-3* (**Table 6**; **Supplementary File S01**). *WRKY33* is required for normal resistance both to necrotrophic fungal pathogens and to abiotic stresses such as salinity and heat (Zheng et al., 2006; Jiang and Deyholos, 2008; Liu et al., 2015; Zhou et al., 2015). *WRKY25* and *WRKY26* also promote resistance to heat stress (Li et al., 2011). However, WRKY genes upregulated by *LGOoe* also have negative or complex effects on defense responses. *wrky53* mutants show increased resistance to *Ralstonia solanacearum*, but decreased resistance to *Pseudomonas syringae* (Murray et al., 2007; Hu et al., 2008), and both *WRKY25* and *WRKY48* negatively regulate resistance to the bacterial pathogen *Pseudomonas syringae* (Zheng et al., 2007; Xing et al., 2008). WRKY51 functions in the salicylic acid pathway to inhibit the jasmonic acid pathway in response to low oleic acid, and thus to inhibit basal resistance to the necrotrophic fungus *Botrytis cinerea* (Gao et al., 2011).

**Table 6:** Heat map of WRKY transcription factors and PR genes.

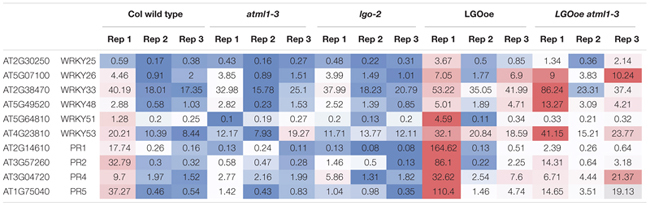

*Heat maps (red high, blue low) showing the expression values (TPM) for WRKY transcription factors and PR genes significantly upregulated by *LGOoe*. The heat map was created for each gene, and is not global. Note that replicate 1 had higher levels of expression of these genes in Col_WT, indicating that some defense response was occurring in replicate 1 even though no infection was visible. Note that, in all cases, these genes were much more highly expressed in *LGOoe* replicate 1, indicating that their expression was also regulated by LGO.*

After the plant activates defense responses at the local site of infection, it also sends a signal to activate *regulation of systemic acquired resistance* [GO:0010112] (SAR), increasing defense responses throughout the plant to protect undamaged tissues from pathogen invasion (Pieterse et al., 2009) (**Table 5**; **Supplementary Files S08** and **S13**). PR genes, which generally encode antimicrobial proteins, are associated with SAR, and *PR1*, *PR2*, *PR4*, and *PR5* are all significantly upregulated in *LGOoe* versus *lgo-2* (**Table 6**; **Supplementary Files S01** and **S08**) (Van Loon et al., 2006). Interestingly, these PR genes were all significantly upregulated in Col_WT replicate 1 versus replicates 2 and 3, but their upregulation was significantly stronger in *LGOoe* replicate 1 plants than in wild type Col_WT replicate 1, indicating that they were regulated by both environment and genotype (**Table 6**; **Supplementary File S01**). No infection was observed in replicate 1 plants; however, given the transcriptomic results, replicate 1 may have had a minor infection that was not visible. In addition, biotic interactions with beneficial microorganisms cause *induced systemic resistance* [GO:0009682] to pathogens through jasmonic acid and ethylene signaling (Pieterse et al., 2009); this function is associated with 10 genes upregulated by *LGOoe* in the absence of ATML1 (*LGOoe atml1-3* versus *atml1-3*; **Figure 4A**; **Table 3**; **Supplementary File S06**).

### Glucosinolate Biosynthesis Is Upregulated by LGO Independently of ATML1

As part of the defense response, genes involved in *glucosinolate biosynthetic process* [GO:0019761] are significantly overrepresented among the genes upregulated by *LGOoe* in the absence of ATML1 (**Figure 4A**; **Table 3**). Glucosinolates are defensive secondary metabolites produced in Brassicaceae species, including *Arabidopsis*, that are responsible for their mustard flavor (Ratzka et al., 2002; Celenza et al., 2005). When the plant tissue is disrupted (e.g., because an insect eats the plant), the damage exposes glucosinolates to myrosinase enzymes that cleave them to make toxic and reactive compounds including nitriles, isothiocyanates, and thiocyanates (Wittstock and Halkier, 2002). In the intact plant, glucosinolates and myrosinases are compartmentalized so that they do not come into contact (Burow et al., 2007), with myrosinases accumulating in distinct cell types from glucosinolates (Koroleva et al., 2000; Thangstad et al., 2004; Li and Sack, 2014). There are three types of glucosinolates: aliphatic, aromatic and indolic (Wittstock and Halkier, 2002). *Indole glucosinolate metabolic process* [GO:0042343] was specifically overrepresented among genes that are more strongly expressed in *LGOoe atml1* versus *atml1-3* sepals (**Table 3**). Glucosinolates contain sulfur, and the sulfur metabolism GO terms *thiosulfate sulfurtransferase activity* [GO:0004792] and *hydrogen sulfide biosynthetic process* [GO:0070814] were both significantly overrepresented among genes upregulated in *LGOoe atml1* versus *atml1-3* (**Table 3**) (Kopriva et al., 2015). In addition to its role in defense against herbivores, the glucosinolate biosynthetic pathway is required for *defense response by callose deposition in the cell wall* [GO:0052544] at the site of pathogen infection (Clay et al., 2009), which was associated with genes upregulated in *LGOoe atml1* versus *atml1-3*.

**FIGURE 5:**
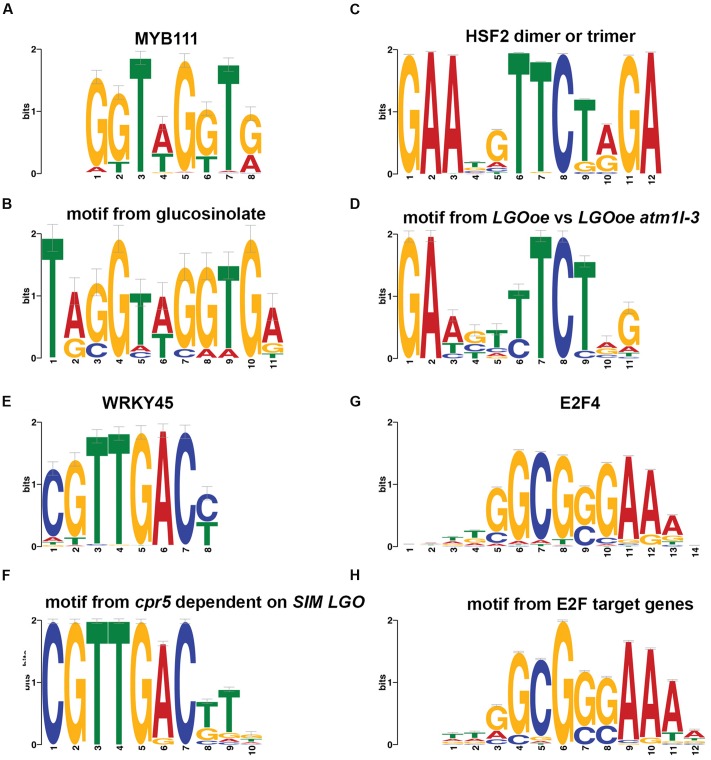
**Promoter motifs.** **(A,B)** A motif found in the 500-nt regions 5′-ward from the start codons of genes differentially expressed in our sepal dataset that were associated with the GO term *glucosinolate biosynthetic process* [GO:0019761] **(B)** matched a binding site previously observed *in vitro* for MYB111 **(A)** (Franco-Zorrilla et al., 2014). *MYB51* was upregulated by *LGOoe*, and is likely to have a similar binding site. **(C,D)** A motif found in the 500-nt regions 5′-ward from the start codons of genes differentially expressed in *LGOoe* versus *LGOoe atml1-3* **(D)** matched with a binding site previously observed *in vitro* for human HSF2 dimers or trimers **(C)** (Jolma et al., 2010). **(E,F)** A motif in the 500-nt regions 5′-ward from the start codons of genes differentially expressed in *cpr5 versus cpr5 sim lgo* **(F)** matched a binding site previously observed *in vitro* for WRKY45 **(E)** (Franco-Zorrilla et al., 2014). This motif was also significantly overrepresented among all 1,341 genes with significant differences in sepal expression between genotypes. **(G,H)** A motif in the 500-nt regions 5′-ward from the start codons of known E2F target genes **(H)** matched a binding site previously observed via ChIP-seq for human E2F4 **(G)** (Kulakovskiy et al., 2016). This motif was significantly underrepresented among all 1,341 differentially expressed sepal genes.

*Regulation of glucosinolate biosynthetic process* [GO:0010439] was significantly associated with genes upregulated in *LGOoe atml1-3* versus *atml1-3* (**Table 3**). The transcription factor gene *MYB51* (also known as *HIGH INDOLIC GLUCOSINOLATE1*, or *HIG1*) regulates indolic glucosinolate biosynthesis (Gigolashvili et al., 2007) and was significantly upregulated in *LGOoe* versus Col_WT, *lgo-2* or *atml1-3* sepals (**Supplementary File S01**). Although both *MYB34* and *MYB122* regulate indole glucosonolate biosynthesis together with *MYB51* (Celenza et al., 2005; Frerigmann and Gigolashvili, 2014; Frerigmann et al., 2016), these genes were not differentially expressed in our RNA-seq data. We observed 10 genes that were upregulated in *LGOoe atml1-3* versus *atml1-3* and that were associated with *glucosinolate biosynthetic process* [GO:0019761]. To determine whether *MYB51* or its paralogs might regulate these 10 genes, we searched for short DNA motifs (6–12 nt long) that were significantly overrepresented in the 500-nt 5′-flanking promoter regions of these 10 genes (regions ranging from 1 to 500 nt 5′-ward of their start codons). We selected a 500-nt sequence range for motif prediction because a majority of conserved non-coding DNA elements (which are likely to include *cis*-regulatory sequences) have been shown to reside within 0.5 kb of the 5′ ends of *Arabidopsis* genes (Haudry et al., 2013). This search identified a motif (5′-TAGGTAGGTGA-3′) that most closely resembled one of two alternative binding sites for MYB111 (Franco-Zorrilla et al., 2014) (**Figures 5A,B**; **Supplementary File S14**). To determine the specificity of this motif, we then used it to search the 500-nt 5′-flanks for all 27,416 protein-coding genes in the *Arabidopsis* genome: this rediscovered 9 out of the 10 original genes in which we had found the motif, along with 317 other genes in *Arabidopsis*. Thus, the motif was found 75 times more frequently in the *LGOoe*-upregulated glucosinolate biosynthetic genes than in the *Arabidopsis* genome as a whole (*p* = 2.2 ⋅ 10^-16^). These data are consistent with the hypothesis that *LGOoe* upregulates *MYB51*, whose product in turn upregulates the glucosinolate biosynthesis transcriptional network in sepals.

### Response to Heat Is Upregulated in *LGOoe* Sepals in an ATML1-Dependent Manner

Although there were few genes upregulated by *LGOoe* in an ATML1-dependent manner, we still investigated their functions. To identify the functions of genes upregulated by *LGOoe* that required ATML1 for upregulation, we examined GO terms associated with genes more strongly expressed in *LGOoe* than in *LGOoe atml1* sepals (**Figure 4A**; **Table 4**; **Supplementary Files S07** and **S12**). Two of the defense responses upregulated by *LGOoe* also depended on ATML1. *Response to hydrogen peroxide* [GO:0042542] depended entirely on ATML1; no genes in that GO term were differentially expressed in *LGOoe atml1-3* versus *atml1-3* (**Figure 4A**; **Table 4**; **Supplementary File S07**). *Response to heat* [GO:0009408] and *response to high light intensity* [GO:0009644] were highly overrepresented among genes upregulated in *LGOoe* versus *LGOoe atml1*, relative to all genes in the genome (**Figure 4A**; **Table 4**; **Supplementary File S07**). Searching for short DNA motifs in the 500-nt 5′-flanks of genes more strongly expressed in *LGOoe* than *LGOoe atml1*, we identified one motif (5′-GAAGTTTCTGG-3’) that matched the binding site *in vitro* for human heat shock factor (HSF) dimers or trimers (**Figures 5C,D**; **Supplementary File S14**) (Jolma et al., 2010). The *Arabidopsis* HSF gene *ATHSF4* (AT4G36990) is upregulated in *LGOoe* versus *atml1-3* or *lgo-2* (**Supplementary File S01**). Thus, ATML1 contributes to some defense responses and environmental responses driven by *LGOoe*, and part of this contribution may be indirectly affected via ATHSF4.

### Overlap of Genes Upregulated by LGOoe With Genes Upregulated by the Constitutive Defense Response Mutant *cpr5* in a SIM- and LGO-Dependent Manner

Consistent with our results that *LGOoe* promotes expression of defense response genes, it has recently been shown that LGO/SMR1 and SIM play a non-canonical role in effector-triggered immunity (Wang et al., 2014). Wang et al. showed that a constitutive defense response found in plants mutant for the gene *constitutive expresser of pathogenesis-related gene5* (*cpr5*) was lost in *cpr5 sim lgo* triple mutants (Wang et al., 2014). They further found that most of the genes differentially expressed between *cpr5* and wild type were dependent on SIM and LGO; in other words, these genes were no longer differentially expressed in *cpr5 sim lgo* compared to wild type. From their published data, we identified 698 genes differentially expressed in *cpr5* versus *cpr5 sim lgo*. Comparing them with 392 genes more strongly expressed in *LGOoe* than in *lgo-2* sepals, we found that 151 genes overlapped, a 15-fold higher overlap than would have been expected by chance (*p* < 2.2 ⋅ 10^-16^). To determine what biological functions might be mediated in common by the two gene sets, we identified GO terms disproportionately associated with the 698 genes differentially expressed in *cpr5* versus *cpr5 sim lgo* (**Supplementary File S15**) and compared them with the GO terms associated with *LGOoe*-upregulated genes (**Supplementary Files S06**–**S08**); this revealed substantial overlap, including *response to chitin* [GO:0010200], *response to wounding* [GO:0009611], *systemic acquired resistance* [GO:0009627], *defense response* [GO:0006952], *defense response to fungus* [GO:0050832], *plasmodesma* [GO:0009506], and *induced systemic resistance* [GO:0009682] (**Figure 4B**; **Supplementary File S15**). To detect possible *cis*-regulatory elements that might mediate these common functions, we searched the 500-nt 5′-flanks of the 698 *cpr5*-upregulated genes for non-coding DNA short motifs, and found a motif (5′-CGTTGACTTG-3′) that matched the binding site for WRKY45 (Franco-Zorrilla et al., 2014) (**Figures 5E,F**). Using this motif to scan the 500-nt 5′-flanks of all 27,416 protein-coding genes in *Arabidopsis*, we found it to be present 1.9-fold more often among all 1,341 genes expressed in sepals with significantly genotype-altered expression than it was present genomewide (47 genes, sepalwide; 506 genes, genomewide; *p* = 6.2 ⋅ 10^-5^; **Supplementary File S14**). For the 392 genes more strongly expressed in *LGOoe* than in *lgo-2* sepals, the overlap was 24 genes, 3.3-fold higher than the genomewide rate (*p* = 4.8 ⋅ 10^-7^). As noted above, WRKY transcription factors play important roles in defense response (Pandey and Somssich, 2009) and several WRKY transcription factors were significantly upregulated by LGO overexpression (**Table 6**; **Supplementary File S01**). Hamdoun et al. (2015) have recently shown that transcriptional activation of defense response primarily requires LGO, with SIM having only a minor role, due to differences in their expression patterns. The overlap of genes upregulated by *LGOoe* with SIM- and LGO-dependent *cpr5*-driven genes expressed provides independent evidence that genes driven by transgenic *LGOoe* in the sepal are indeed regulated by LGO *in vivo*.

### LGO Does Not Regulate Transcription in the Mature Sepal Directly Through E2Fs

One mechanism through which LGO could regulate transcription is by modulating the activity of E2F cell-cycle transcription factors. Furthermore, Wang et al. demonstrated that *e2f* mutants suppress constitutive defense response in *cpr5* mutants similarly to *lgo sim*, suggesting that LGO and SIM promote defense response through their regulation of the E2F pathway (Wang et al., 2014). Because Wang et al. (2014) found that several genes known to be regulated by E2F were upregulated in *cpr5* mutants (Vandepoele et al., 2005), we tested whether there was overlap between the 1,341 genes expressed in sepals with significantly genotype-altered expression and the known set of 180 E2F target genes in *Arabidopsis* (Vandepoele et al., 2005). Unexpectedly, we found that the overlap between known E2F target genes and differentially expressed sepal genes was almost non-existent (only 4 genes, 2.2-fold lower than expected by chance; *p* = 0.125; AT1G05490, chromatin remodeling 31; AT3G57260, BETA-1,3-GLUCANASE 2; AT4G14365, XBAT34; and AT5G49520, ATWRKY48). We determined the GO terms associated with the known 180 E2F target genes, and found them to be generally associated with *DNA replication initiation* [GO:0006270] and *cell cycle* [GO:0007049] (**Supplementary File S15**); however, when we compared them with GO terms associated with *LGOoe*, we observed no overlap at all (**Figure 4B**; **Tables 3**–**5**; **Supplementary Files S06**–**S08**). Finally, we searched for short DNA motifs associated with the 500-nt 5′-flanks of the 180 known E2F target genes and recovered a motif (5′-TTGGCGGGAAAA-3′), that matched the binding site for human E2F4 (**Figures 5G,H**) (Kulakovskiy et al., 2016). Scanning all 27,416 protein-coding genes for occurrences of this motif in their 500-nt 5′-flanks yielded a set of 786 predicted E2F targets genomewide. The overlap of these predicted E2F target genes to the 1,341 differentially expressed sepal genes was 2.0-fold lower than expected by chance, more significantly than with known E2F target genes (19 genes; *p* = 7.4 ⋅ 10^-4^; AT1G03080, NET1D; AT1G07970; AT1G14420; AT1G15825; AT1G15830; AT1G54215; AT2G17840, EARLY-RESPONSIVE TO DEHYDRATION 7 [ERD7]; AT2G36570, PXC1; AT2G42890, MEI2-like 2; AT2G43865; AT3G19620; AT4G05553; AT4G13000; AT4G23230, CRK15; AT4G34440, AtPERK5; AT4G35610; AT5G25930; AT5G54960, pyruvate decarboxylase-2; AT5G64510, and tunicamycin induced 1 [TIN1]). These results indicate that genes driven by *LGOoe* in differentiated sepals are not regulated directly through E2F transcription factors and the standard cell cycle pathway.

### KRP5 Target Genes Do Not Overlap With Genes Differentially Expressed in LGOoe Sepals

Jégu et al. (2013) identified 264 genes bound by KRP5 using ChIP-seq. To examine the specificity of our results we examined the overlap between these 264 genes and the 1,340 genes that we found to be significantly differentially expressed (up- or down- regulated) in response to overexpression of LGO in some genotype (*LGOoe*). We found that only six genes belonged to both sets; this represents a modestly significant decrease of KRP5 targets in our *LGOoe*-regulated gene set. Specifically, this is a 2.1-fold underrepresentation, compared to the overlap of 13 genes that would be expected by chance; it has a *p*-value of 0.05 by a two-sided exact binomial test with a 99% confidence interval. We conclude that there is no evidence that KRP5 preferentially binds our *LGOoe*-regulated genes; indeed, the evidence suggests that it may be less likely to do so than one would expect randomly.

### Limited Similarity between LGOoe Sepals and Mature Trichomes

We looked for similarities between the genes upregulated in sepals by *LGOoe*, where the sepal is covered with giant cells (**Figures 1H,I**), and genes specifically expressed in mature trichomes, because sepal giant cells and trichomes are both highly endoreduplicated cell types. However, each of two published mature trichome gene sets were only modestly enriched for overlaps with the 1,341 genes differentially expressed between different sepal genotypes: out of 1,143 trichome-specific genes from Jakoby et al. (2008), only 82 overlapped with the sepal genes (1.5-fold more than expected by chance; *p* = 7.8 ⋅ 10^-4^); and out of 788 trichome-specific genes from Marks et al. (2009), only 84 overlapped with the sepal-expressed genes (2.2-fold more than expected by chance; *p* = 8.4 ⋅ 10^-11^). Moreover, when we analyzed GO terms associated with two different sets of genes found to be specifically expressed in mature trichomes (**Supplementary File S15**) (Jakoby et al., 2008; Marks et al., 2009), few overlapped with the GO terms associated with *LGOoe*-driven genes (**Figure 4B**; **Supplementary Files S06**–**S08**). However, metabolic pathway analysis has previously shown that glucosinolate biosynthesis is upregulated in mature trichomes (Jakoby et al., 2008) and we find glucosinolate biosynthesis upregulated in *LGOoe* sepals, indicating one important similarity.

## Discussion

Motivated by evidence that cell cycle regulators can directly and indirectly control transcription, we used transcriptomics to determine to what extent the CKI LGO affects gene expression in mature *Arabidopsis* sepals. While the primary role of LGO is to promote endoreduplication at early stages of sepal development (**Figure 1**), we found that in mature sepals overexpression of *LGO* throughout the sepal epidermis mainly increased expression of genes associated with defense responses to fungi, insects, and bacteria. Notably, the indolic glucosinolate biosynthesis pathway, whose products defend against insect herbivores, was upregulated by overexpression of *LGO*. Other genes that were upregulated in the sepal by *LGO* overexpression, but that are currently uncharacterized, may encode new components of the defense response. The RNA-seq data generated here provide a high-quality, public transcriptomic resource for future study of the interaction between cell cycle genes and the defense response in *Arabidopsis*.

Our results support the emerging connection between the cell cycle and plant defense response (Bao and Hua, 2015). Many of the SIAMESE related CKIs are induced in response to pathogens or abiotic stress (Peres et al., 2007). For example, in rice, the *EL2* gene is rapidly induced upon treatment with *N*-acetylchitoheptaose (a biotic elicitor for phytoalexin biosynthesis) or with bacterial flagellin (Minami et al., 1996; Che et al., 2000). Furthermore, Bao et al. (2013) recently found that an activation-tagging allele upregulating expression of *OMISSION OF THE SECOND DIVISION* (*OSD1*), which encodes a negative regulator of the anaphase-promoting complex/cyclosome (APC/C), showed both increased immunity to a bacterial pathogen and increased expression of resistance (R) genes. Our results similarly show that overexpression of the cell cycle regulator LGO induces expression of many classes of defense response genes. Wang et al. (2014) have previously shown that LGO and SIM participate in the defense response, that *lgo sim* double mutants are abnormally susceptible to infection with *Pseudomonas syringae*, and that many genes upregulated in the *cpr5* constitutive defense response mutant were no longer upregulated in the *cpr5 lgo sim* triple mutant. In other words, the *cpr5*-activated genes characterized by Wang et al. (2014) depended on LGO and SIM for expression. Hamdoun et al. (2015) have subsequently demonstrated that LGO is more strongly required for the defense response than its paralog SIM. We have found that genes upregulated by the *cpr5* mutation in a SIM- and LGO-dependent manner disproportionately overlap with genes that are upregulated when *LGO* is overexpressed in sepals, indicating that LGO is not only necessary but sufficient to drive the expression of defense response genes. Although our data is based on *LGOoe*, and thus may not reflect the endogenous functions of the *LGO* gene, the overlap with genes no longer upregulated in the *cpr5 lgo sim* triple mutant increases credence that LGO directly or indirectly regulates these genes. Conversely, we find that E2F transcription factors, which have been demonstrated to act downstream of LGO and SIM in *cpr5* plants, are unlikely to be direct transcriptional activators of defense genes in *LGOoe* sepals. In our RNAseq data the expression of the defense response genes is highly variable, although significant differences in expression due to *LGOoe* were detected within the noise. Some of the variability may be due to unseen pathogen attack on the plants from which the RNAseq samples were isolated. It is also possible that expression of some pathogen response genes is further enhanced by *LGOoe*.

The mechanism through which overexpression of LGO regulates gene activity requires further investigation, but the transcriptomic results themselves provide some insight. In *LGOoe* plants, a *LGO* transgene is driven by the *ATML1* promoter, which is expressed throughout the epidermis of sepals from early stages through maturity (Lu et al., 1996; Sessions et al., 1999; Roeder et al., 2010). Therefore, *LGO* overexpression likely drives downstream genes through more than one mechanism at more than one stage of sepal development. This is further supported by our finding that the transcription factors *MYB51*, *ATHSF4*, and *WRKY33* are upregulated in *LGOoe* sepals, along with putative downstream targets encoding components of glucosinolate biosynthesis, heat shock response, and defense response, and that these downstream targets share distinct *cis*-regulatory motifs for binding transcription factors (for MYB, HSF, and WRKY) in their proximal promoter regions.

Based on similarity of the *LGOoe* sepal phenotype to *ATML1::KRP1* sepals, in which live imaging has shown induction of endoreduplication in sepal primordia to form giant cells (Roeder et al., 2010), induction of endoreduplication and formation of giant cells occurs at early stages of sepal development (stages 6–10) before our assay of the transcriptome at stage 12, which explains why we do not observe significant differences in expression of any cyclins or CDKs. In this context, there are several ways one might explain LGO’s effects on gene activity. First, it might act through endoreduplication. In the budding yeast *Saccharomyces cerevisiae*, it has been shown that a change in ploidy alone is sufficient to alter gene expression (Galitski et al., 1999; Wu et al., 2010). However, if endoreduplication alone were responsible for the differential expression of genes in *LGOoe* sepals, we would expect substantial overlap with the genes expressed in mature trichomes, another highly endoreduplicated cell type. In fact, we observed modest overlap between *LGOoe* genes and mature trichome genes, suggesting that endoreduplication alone is not a sufficient mechanism to explain all of the gene expression in *LGOoe* sepals, although it may explain some transcriptomic effects. Endoreduplication in *LGOoe* sepals may regulate the expression of genes indirectly in that endoreduplication causes the formation of giant cells, which in turn means there are fewer cells available to enter the stomatal patterning pathway. The expression of stomatal patterning genes *FAMA*, *ERECTA-LIKE2* (*ERL2*), and *STOMATAL CARPENTER1* (*SCAP1*) is significantly downregulated in *LGOoe atml1-3* sepals, which have very few stomata, relative to *lgo-2* sepals, which have many stomata. However, ATML1 is also known to promote stomatal development (Peterson et al., 2013; Takada et al., 2013), so this may be due to a combination of factors. Second, because LGO regulates the cell cycle, and because E2F has been implicated in LGO-dependent transcriptional activation of defense-response genes in *cpr5* plants, one might expect that LGO would regulate transcription in sepals via E2F transcription factors. However, the genes in our gene set have essentially no overlap with E2F target genes (Vandepoele et al., 2005). They also have a significantly lower frequency (compared to the genome as a whole) of genes with an E2F binding motif in their 500-nt 5′ flanks, suggesting that direct mediation of transcription by E2Fs is unlikely. In our analysis, RNA-seq was performed on RNA isolated from mature sepals after division has ceased; the lack of cell cycle genes in our *LGOoe*-driven gene sets is thus not unreasonable. It is likely that transcriptional effects of LGO regulating E2F were present at earlier stages of development. Although we do not find evidence of E2F targets in our dataset, it is possible that the changes in gene expression caused by *LGOoe* occur indirectly through the cell cycle via CDKs regulating other transcription factors and RBR. CDKA;1 has been shown to phosphorylate HEAT SHOCK FACTOR1 (HSF1) modulating the DNA binding activity *in vitro* (Reindl et al., 1997) and we found that genes involved in response to heat were upregulated by *LGOoe* in an ATML1-dependent manner. Third, previous results show that LGO can interact with the bZIP69 transcription factor and the chromatin remodeler BRAHMA (Van Leene et al., 2010), suggesting that LGO could play a more direct role in regulating transcription. In tomato, a set of protein–protein interactions was identified via yeast two-hybrid that included a SIAMESE-related CKI (SIP4), two 14-3-3 proteins, the SPAK kinase, the SPGB bZIP transcription factor, and the phosphatidylethanolamine binding protein SELF PRUNING (Pnueli et al., 2001). In plants, this family of phosphatidylethanolamine binding proteins includes many important developmental regulators including FLOWERING LOCUS T (FT) and TERMINAL FLOWER (TFL), which control flowering time and inflorescence meristem determinacy respectively (Alvarez et al., 1992; Corbesier et al., 2007). Notably, *phosphatidylethanolamine binding* [GO:0008429] is significantly overrepresented among genes upregulated by *LGO* overexpression that do not depend on ATML1 (**Table 3**); for instance, the genes *FLOWERING LOCUS T* (*FT*) and *BROTHER OF FT AND TFL* (*BFT*) are significantly more strongly expressed in *LGOoe atml-3* than in *atml1-3* sepals (**Supplementary File S01**). Further investigation of possible transcriptional complexes upon which LGO might act directly remains for future research.

*LGO* overexpression throughout the epidermis not only produces cells that cannot be phenotypically distinguished from giant cells (**Figure 1**), but also induces the expression of defense response genes. Transcriptional responses to the environment can be highly cell-type specific (Dinneny et al., 2008). As the epidermis is the first line of defense against pathogens, it would not be surprising to find that epidermal cell types have specific defensive roles mediated by upregulation of epidermis-specific batteries of genes. For example, the myrosinases that cleave glucosinolates into toxic and reactive compounds upon tissue disruption (i.e., herbivore predation) are expressed specifically in guard cells and myrosin idioblast cells (Thangstad et al., 2004; Li and Sack, 2014), which would biologically complement upregulation of glucosinolate biosynthesis in giant cells.

In conclusion, our findings support the hypothesis that *LGO* itself can directly or indirectly drive sepal epidermal cells to specific states of gene expression; in particular, they are consistent with recent findings showing *LGO* to be necessary for transcriptional activation of defense genes in *Arabidopsis*.

## Materials and Methods

### Plants

Plants were grown in pots of Lambert General Purpose Mix LM111 soil in Percival growth chambers in continuous light at 22°C to minimize circadian effects on the transcriptome. The Columbia-0 (Col-0) accession of *Arabidopsis* was used as wild type (Col_WT). The *lgo-2* (SALK_033905) and *atml1-3* (SALK_033408) alleles have been described previously (Roeder et al., 2010, 2012). Plants overexpressing *LGO* in the epidermis [*LGOoe*, i.e., plants carrying the transgene *ATML1p::LGO*, encoded by the plasmid pAR178 as described previously (Roeder et al., 2010)] were generated by *Agrobacterium*-mediated transformation of Col-0 wild type plants with pAR178 and selection of a line segregating 3:1 resistant:sensitive phenotypes to the herbicide Basta. The *LGOoe atml1-3* plants were generated by crossing, genotyping for the *atml1-3* mutant, and assessing homozygosity of *LGOoe* based on phenotypic segregation in subsequent generations.

### Scanning Electron Microscopy (SEM)

Scanning electron microscopy was performed as described in Roeder et al. (2003). Stage 12 flowers were fixed in FAA (3.7% formaldehyde, 50% ethanol, 5% acetic acid) for 4 h with 15 min vacuum infiltration. Samples were dehydrated through an ethanol series (50, 60, 60, 60, 90, 95, 100%) and critical-point dried with a BAL-TEK CPD030. Sepals were dissected and mounted on stubs, sputter coated with platinum palladium and imaged with a Tescan Mira 3 FESEM scanning electron microscope in the Cornell Center for Materials Research.

### RNA Isolation

For each genotype (wild type Col-0, *lgo-2*, *atml1-3*, *LGOoe*, and *LGOoe atml1-3*), three biological replicate samples were analyzed. The genotypes within one replicate were grown simultaneously together in a flat. Replicates 1, 2, and 3 were grown at different times in different flats. For each sample, 250 stage 12 sepals were dissected and immediately frozen in liquid nitrogen; sepals were staged according to Smyth et al. (1990). Sepals were dissected in the growth chamber where the plants were growing to minimize transcriptional changes during the dissection process. Sepals were dissected between 5:00 PM and 8:20 PM to minimize circadian effects on the transcriptome. RNA was purified using the QIAGEN RNeasy Plant Mini kit (Qiagen Cat# 74904) according to the manufacturer’s instructions. RNA was treated with DNAse I amplification grade (Invitrogen Cat# 18068015) according to manufacturer’s instructions before library preparation.

### qPCR Measurement of LGO Expression

For each genotype (*LGOeo*, wild type Col, and *lgo-2*), three biological replicates were analyzed. For each sample, RNA was isolated from three pooled inflorescences including flowers up to stage 12 using the QIAGEN RNeasy Plant Mini kit (Qiagen Cat# 74904) according to the manufacturer’s instructions. One microgram of RNA for each sample was treated with DNAse I amplification grade (Invitrogen Cat# 18068015) according to manufacturer’s instructions. First strand cDNA was synthesized with oligo dT using Superscript II (Invitrogen Cat#18064014). Real-time PCR was performed using SYBR Green Master Mix (Roche Cat# 4707516001) on a Roche *LightCycler* 440 system with three technical replicates. LGO was quantified using primers oHM63 (5′-AGA ACA CAA GAT TCC CGC CG-3′) and oHH64 (5′-ACG GAG GAG AAG AAA CGG TC-3′). *ROC1* (AT4G38740) was used as a reference gene to normalize gene expression with primers cyclo-F (5′-CGA TAA GAC TCC CAG GAC TGC CGA-3′) and cyclo-R (5′-TCG GCT TTC CAG ATG ATG ATC CAA CC-3′). qPCR results were analyzed by the ΔΔCt method. Statistics were calculated on ΔCt values using the *t*-test.

### Generation of RNA-seq Data

RNA-seq library preparation was performed as in Kumar et al. (2012) with modifications for individual sample preparation. Briefly, mRNA was isolated with Dynabeads oligo dT beads (Invitrogen Cat# 61006) and fragmented for 5 min at 70°C with the PCR block lid at 105°C using Fragmentation buffer (Ambion Cat# AM8740). First strand cDNA was synthesized with random hexamers (Invitrogen Cat# 48190011) using Superscript II (Invitrogen Cat#18064014) and the second strand was synthesized with DNA Pol I (Fermentas Cat #EP0041) and RNAse H (Invitrogen Cat# 18021071). End repair was conducted with NEBNext End repair enzyme Mix (NEB Cat# E6050S) and Klenow DNA polymerase (NEB Cat# M0210S), A tailing with Klenow 3′ to 5′ exonuclease (Fermentas Cat# EP0421), and ligation of adapters containing 3 bp in read barcodes with Mighty Mix Ligase (Clonetech TAK6023). The library was size-selected using Agencourt AmPure Beads (Beckman Coulter A63880). PCR enrichment was conducted with primers PE 1.0 (5′-AAT GAT ACG GCG ACC ACC GAG ATC TAC ACT CTT TCC CTA CAC GAC GCT CTT CCG ATC* T-3′) and PE 2.0 (5′-CAA GCA GAA GAC GGC ATA CGA GAT CGG TCT CGG CAT TCC TGC TGA ACC GCT CTT CCG ATC* T-3′) for 15 cycles using Phusion polymerase (NEB). The entire library was run on a 1.2% agarose gel and size-selected (about 200–500 bp) to remove adapter dimers. Libraries for each replicate were pooled and sequenced on two lanes using rapid run settings on an Illumina HiSeq 2000 to generate single-end 51-nt reads at the Cornell University Biological Resource Center Genomics Facility.

### Quality-Filtering and Barcoding RNA-seq Reads

We quality-filtered our raw 51-nt RNA-seq reads in two steps. First, we used a custom Perl script (*quality_trim_fastq.pl*, provided in **Supplementary File S16**) to remove reads that failed CHASTITY testing, to trim the 51st nucleotide off of our raw reads, and to censor any aberrant reads under 50 nt in length; the arguments used were “*quality_trim_fastq.pl -i -* [i.e., *zcat* input stream] *-q 33 -u 50 -o* [output FastQ file].” Second, we used Trimmomatic 0.33 (Bolger et al., 2014) to remove adapter sequences, to trim off unreliable residues, and to censor any reads that such trimming reduced to ≤49 nt; the arguments used were “*java -jar trimmomatic SE -threads 7 -phred33* [input FastQ file] [output FastQ file] *ILLUMINACLIP:[adapter sequences FastA file]:2:30:10 LEADING:3 TRAILING:3 SLIDINGWINDOW:4:15 MINLEN:50*”. Adapter sequences provided to Trimmomatic are listed in **Supplementary File S17**. Third, having stringently quality-filtered our reads, we split them via barcodes into discrete RNA-seq data sets with *fastx_barcode_splitter.pl* from FastX-Toolkit 0.0.14^1^^2^^3^); the line command format and arguments used were “*cat* [input file] | *fastx_barcode_splitter.pl –bcfile* [barcode file] *–bol –mismatches 0 –prefix* [specific data output prefix] *–suffix “.fq”* ”. Barcodes provided to *fastx_barcode_splitter.pl* are listed in **Supplementary File S18**. Fourth, having sorted the reads by barcodes, we trimmed off their 5′-most four nucleotides *in silico* with *fastx_trimmer* from FastX-Toolkit 0.0.14; the arguments used were “*-f 5 -i* [input reads] *-o* [output reads].” We trimmed off the 5′-most four nucleotides in order to remove not only the 3-nt barcodes but also a fourth non-cDNA residue added during the construction of Illumina RNA-seq libraries. After this quality-filtering, we obtained between 7,615,242 and 33,257,356 reads per replicate (48,448,184 to 82,408,319 reads per genotype; **Supplementary File S19**). We used the final reads (quality-filtered, barcode-sorted, and barcode-trimmed) for all further RNA-seq analyses, and archived them in the NCBI Sequence Read Archive database (SRA^4^).

### Analysis of RNA-seq Data

To enable mapping of RNA-seq reads to *Arabidopsis* genes, we constructed a transcript index with RSEM 1.2.21 (Li and Dewey, 2011). This index primarily contained 27,416 protein-coding sequences from the TAIR10 release of the *Arabidopsis* genome database (Berardini et al., 2015). As negative control sequences, it also included *Arabidopsis* ribosomal ncRNAs, a GFP transgenic coding sequence, and the paired-end adapter sequences used in our RNA-seq libraries. Sources for all of these sequences are given in **Supplementary File S20**. We computed RNA-seq expression values for *Arabidopsis* genes by mapping quality-filtered, barcode-split and -trimmed read sets to our transcript index with bowtie2 version 2.2.5 (Langmead and Salzberg, 2012), SAMTools 1.2 (Li et al., 2009), and *rsem-calculate-expression* in RSEM 1.2.21. For each RNA-seq data set, the numbers of reads mapped to a unique site in the transcriptome, mapped to two or more sites, or that were unmapped are listed in **Supplementary File S19**. The arguments used for RSEM included “*rsem-calculate-expression –bowtie2 –calc-pme –phred33-quals –calc-ci –ci-credibility-level 0.99 –fragment-length-mean* [mean insert size in nt] *–fragment-length-sd* [standard deviation of insert sizes in nt].” Mean insert sizes for each RNA-seq library were determined by reading the library’s peak insert size from its Bioanalyzer report, and subtracting 127 nt to account for linker sequences; the standard deviation for each library’s insert size was estimated visually from its Bioanalyzer report by taking the average of size changes that reduced the density of the library inserts by half. Mean sizes and standard deviations for RNA-seq libraries are given in **Supplementary File S19**. For each gene, from the RSEM results for each RNA-seq read set, we extracted the following data: posterior mean estimate of TPM, a normalized measurement of gene expression; the minimum TPM (minTPM) in the 99% confidence interval of expression values computed by RSEM; and the integer portion of the posterior mean estimate of reads mapped to that gene (rounding off decimal fractions in RSEM’s estimate). All of these gene expression data are provided in **Supplementary File S01**. All read sets mapped to *Arabidopsis* genes at high frequencies (83.6–87.2% per replicate), with 46.4–48.8% of reads per replicate mapping to unique sites in the *Arabidopsis* transcriptome (**Supplementary File S19**).

To determine whether a given *Arabidopsis* gene was actually expressed in a given RNA-seq data set (i.e., if its measured expression was above background noise), we compared the minTPM for that gene to the nominal expression value (in TPM, not minTPM) for the coding sequence of transgenic GFP. In reality, none of the sources of RNA-seq data in our work had any GFP transgene, let alone an active one. Therefore, the expression level computed by RSEM for GFP in a given RNA-seq data set was a reasonable empirical measurement of background noise. Thus, if the minTPM computed for an actual *Arabidopsis* gene in a given RNA-seq data set was greater than the TPM computed for GFP, we classified that gene as actually being expressed. We detected expression of 20,241 genes in wild-type sepals (73.8% of all genes), and expression of 1,358 more genes (5.0%) in sepals of other genotypes (**Supplementary File S21**), with expression levels per gene ranging from ∼92,000 to 0.07 TPM.

To determine which changes of gene expression between batches or genotypes were statistically significant, we analyzed integral readcounts per gene with DESeq2 version 1.10.1 (Love et al., 2014). We determined significant changes of gene expression between genotypes while controlling for the effects of batches, and also determined significant changes of gene expression between batches (replicates) while controlling for the effects of genotypes. Increases or decreases of gene expression were considered significant if they had a Benjamini–Hochberg-adjusted *p*-value of ≤0.1, i.e., if after correction for multiple hypothesis testing they had a collective false discovery rate (FDR) of ≤0.1 (Noble, 2009). The specific R commands used to generate DESeq2 values for these comparisons are given in **Supplementary File S22**. All instances of significantly changed gene expression are listed in **Supplementary File S01**. To check these results, we also analyzed readcounts per gene with the exactTest function of edgeR version 3.12.1 (Robinson et al., 2010). This gave sets of genes with significantly changed gene expression that were generally smaller than those sets predicted with DESeq2; at the same time, genes predicted by edgeR were almost always also predicted by DESeq2 (**Supplementary File S03**). The specific R commands used to generate edgeR values for these comparisons are given in **Supplementary File S23**. Because the edgeR predictions were essentially a subset of the DESeq2 predictions, we used the DESeq2 predictions for all subsequent analyses.

A heatmap of global gene activity (**Figure 2A**) was drawn with *pheatmap* 1.0.8 in R^5^ with the arguments “*mat, scale = “row”, show_rownames = FALSE, cluster_cols = FALSE, clustering_method = “ward.D2”, width = 6, height = 6, clustering_distance_rows = “correlation”, color = colorRampPalette (c(“darkturquoise”, “black”, “yellow”))(150)*”, using expression data in TPM for the set of 1,341 genes with significant differences in sepal expression between genotypes. The expression values of genes (in rows, with their conditions in column) were centered and scaled, so that relative rather than absolute expression levels would be displayed. These normalized expression values were grouped using an updated version of Ward’s minimum variance method, which is designed to find compact, spherical clusters^6^, with distances between the gene expression profiles being determined by their Pearson correlation. A principal component analysis (PCA) of global gene activity (**Figure 2B**) was generated with DESeq2, from the same readcount data used to produce statistical significances for changes in gene expression. Venn diagrams were drawn in Photoshop CC 2015 based on the data for significantly changed gene expression in **Supplementary File S01**. Heat maps for expression levels of individual genes (**Tables 2** and **6**) were created in Microsoft Excel using conditional formatting for the TPM values.

### Gene Annotations

To provide informative descriptions for the genes listed in **Supplementary File S01**, we obtained annotations from the following sources. Gene aliases were downloaded and extracted from ftp://ftp.arabidopsis.org/home/tair/TAIR_Public_Releases/TAIR_Data_20140331/gene_aliases_20140331.txt. Gene descriptions (including short descriptions, summaries, and computational descriptions of gene function) were downloaded and extracted from ftp://ftp.arabidopsis.org/home/tair/Genes/TAIR10_genome_release/gene_description_20131231.txt.gz. InterPro protein domains encoded by genes were downloaded and extracted from http://www.arabidopsis.org/download_files/Proteins/Domains/TAIR10_all.domains. Gene aliases, gene descriptions, and protein domains were from the TAIR10 release of the *Arabidopsis* genome database (Berardini et al., 2015). Lists of genes encoding transcription factors, and the specific class of transcription factor so encoded, were extracted from *families_data.tbl* (which itself was downloaded and extracted from http://arabidopsis.med.ohio-state.edu/Downloads/AtTFDB.zip) and downloaded and extracted from http://planttfdb.cbi.pku.edu.cn/download/gene_model_family/Ath, being merged into a single annotation set; these two sets of transcription factor annotation data were, respectively, from the databases AtTFDB (Yilmaz et al., 2010) and PlantTFDB (Jin et al., 2014).

### Identifying Previously Published Sets of Genes

We extracted groups of genes from the following previously published analyses, because of their importance to understanding *LGOoe*-driven gene activity in sepals: 710 genes that are significantly more strongly expressed in *cpr5* than in *cpr5 sim lgo* plants (i.e., genes upregulated by *cpr5* in a SIM- and LGO-dependent fashion) (Wang et al., 2014), of which 698 could be identified with protein-coding genes in TAIR10; 181 genes that are likely targets of the transcription factor E2F in *Arabidopsis* (Vandepoele et al., 2005), of which 180 could be identified with protein-coding genes in TAIR10; 1,148 genes with trichome-specific expression, as observed by Jakoby et al. (2008), of which 1,143 could be identified with protein-coding genes in TAIR10; 802 genes with trichome-specific expression, as observed by Marks et al. (2009), of which 788 could be identified with protein-coding genes in TAIR10; and 264 protein-coding genes in TAIR10 that were observed, by Jégu et al. (2013) via ChIP-seq, to be bound by KRP5. Our lists of these gene sets are given in **Supplementary File S24**.

Genes that were significantly more strongly expressed in *cpr5* than in *cpr5 sim lgo* were extracted from the microarray data of Wang et al. (2014)^7^ via the NCBI’s GEO2R portal^8^. We used GEO2R to compare GSM991297, GSM991298, and GS991299 (*cpr5_rep1*, *cpr5_rep2*, and *cpr5_rep3*) to GSM991303, GSM991304, and GSM991305 (*cpr5 sim smr1_rep1*, *cpr5 sim smr1_rep2*, and *cpr5 sim smr1_rep3*); we selected “Submitter supplied” platform annotations, but otherwise ran GEO2R with default settings. The resulting data were exported from NCBI as tab-delimited data tables, and filtered for genes that had any upregulation at all (i.e., any positive value for the logarithm of their expression ratios) with an adjusted *p*-value of ≤0.001.

Likely E2F target genes in *Arabidopsis* were extracted from Supplementary Table S3 of Vandepoele et al. (2005).

Trichome-specific genes were extracted from Supplementary Table S1A of Jakoby et al. (2008) by selecting all genes with a “FC mT/mLwoT” ratio of ≥1 that were also annotated as “trichome-specific” with an asterisk in the table. These genes were defined by Jakoby et al. (2008) as genes with both of the following two traits: they were up-regulated in mature trichomes relative to leaves without trichomes; and they were not expressed in leaves without trichomes.

Trichome-specific genes were extracted from Supplemental Table S1 of Marks et al. (2009) by the following method, derived from the text of Marks et al. (2009). The raw gene expression for wild-type mature trichomes, for mutant *gl3-sst sim* trichomes (with genetic blocks against the differentiation of mature trichomes), and for wild-type shoots were all compared against one another. Any gene that was expressed in wild-type mature trichomes, and that exhibited either ≥3-fold lower expression or no expression at all in either mutant *gl3-sst sim* trichomes or wild-type shoots, was then selected as trichome-specific.

### Gene Ontology Term Analysis

We used *func_hyper* and *func_hyper_refin* in FUNC 0.4.7 (Prüfer et al., 2007) to identify non-redundant GO terms that were statistically overrepresented among groups of protein-coding genes. These groups primarily consisted of gene sets that we determined, in this work, to be significantly up- or down-regulated in responses to changes in genotype or batch (**Supplementary File S01**). However, we also analyzed GO terms associated with previously published groups of genes of relevance to this work (see above).

A key feature of FUNC is that it can use the GO term hierarchy to discount p-values for broad, high-level GO terms if these values arise solely from more specific, low-level GO terms annotating a subset of the genes annotated by a high-level term. The GO term hierarchy was downloaded from the GO consortium at http://archive.geneontology.org/full/2015-12-01/go_201512-termdb-tables.tar.gz. GO terms for protein-coding genes in TAIR10 were extracted from the GO annotation file http://geneontology.org/gene-associations (released on 2/1/2016), and reformatted into FUNC-usable input files with the custom Perl scripts *build_func_boolean.pl* and *qual2func_table_arath.pl* (**Supplementary File S16**). As noted above, each group of genes from our sepal expression data was selected by having an adjusted *p*-value of ≤0.1 and by sharing either increased or decreased expression in response to a given change of conditions. The GO terms associated with each group of genes were then tested against the GO terms associated with the total list of protein-coding genes in the TAIR10 release of the *Arabidopsis* genome database^9^. We first ran *func_hyper* with the arguments “*-i [FUNC-formatted gene set file] -t go_201512-termdb-tables -o [output directory for FUNC analysis]*”, which gave us an initial set of GO terms statistically overrepresented among the gene set by comparison to all genes in the genome, based on the hypergeometric statistical distribution for binary associated comparisons. We then refined these results, in order to select only the most specific GO terms that actually had statistical enrichment among differentially expressed genes while discarding more broad parent GO terms that derived all their significance from more specific child terms, by running *func-refin* (via the automatically generated script *refin-YEAR-MM-DD.sh*) with the arguments “*0.01* [pvalue] *0.01* [pvalue-after-refinement] *1* [minimum number of genes in a subgroup for refinement].” All of the final, refined GO terms for gene groups from our sepal expression data are summarized in **Supplementary File S05**; final, refined GO terms for gene groups from previously published data are summarized in **Supplementary File S15**.

Gene ontology hierarchical graphs were made with AgriGO using Singular Enrichment Analysis (SEA) for *Arabidopsis thaliana* genemodel (TAIR9^10^) with default parameters (Du et al., 2010).

Bar graphs for GO fractions of genes (**Figures 4A,B**) were generated using the *func_hyper* results prior to refinement, which provided gene counts. Specific terms were chosen to illustrate trends. The fraction of genes in the genome associated with a given GO term was calculated by dividing #genes_in_node by #genes_in_root_node. The fraction of genes from each gene set associated with a given GO term was calculated by dividing #genes_with_variable = 1_in_node by #genes_with_variable = 1_in_root_node. Bar graphs were created in Microsoft Excel. Terms that were significantly enriched (raw_p_overrepresentation_of_variable = 1 < 0.01) were marked with an asterisk, and terms that were significantly depleted (raw_p_underrepresentation_of_variable = 1 < 0.01), were marked with a dagger (†).

### Non-coding DNA Motif Prediction and Analysis

We extracted non-coding DNA motifs from the 500-nt 5′-flanking sequences of *Arabidopsis* protein-coding genes (in *TAIR10_upstream_500_translation_start_20101028.fa*, obtained from ftp://ftp.arabidopsis.org/home/tair/Sequences/blast_datasets/TAIR10_blastsets/upstream_sequences/TAIR10_upstream_500_translation_start_20101028) with MEME 4.11.1 (Bailey et al., 2015), using the arguments “*-evt 0.1 -dna -mod zoops -nmotifs 20 -minw 6 -maxw 12 -bfile TAIR10_500nt_trans_markov1-revcomp-maxsize 10000000*”. Before running MEME, we generated the Markov-1 background file *TAIR10_500nt_trans_markov1* with the argument “-m 1”. To produce this background, we extracted and used an all-contigs derivative of *TAIR10_upstream_500_translation_start_20101028.fa* in which non-ACGT residues were censored and their adjacent contigs were split into separate sequences; this was done in order to build the background model with a standard nucleotide alphabet (ACGT). MEME searches were performed on defined sets of genes, rather than all 27,416 protein-coding genes at once. Most of these sets were subsets of the 1,341 genes for which we observed statistically significant changes of gene activity between genotypes; the subsets were selected either by shared GO terms (e.g., *response to heat* [GO:0009408]) or by shared genotypic changes under which expression changed significantly (e.g., *LGOoe atml1-3* versus *atml1-3*, upregulated). In addition, we searched for motifs in a number of previous published gene sets of possible relevance to sepal biology (e.g., E2F target genes and *cpr5*-upregulated, SIM/LGO-dependent genes).

For all motifs discovered by MEME, we used TOMTOM from MEME 4.11.1 (Tanaka et al., 2011) with the argument “*-bfile TAIR10_500nt_trans_markov1*” to check them for similarity to previously identified motifs in the following motif databases distributed with MEME: ARABD, CIS-BP, EUKARYOTE, FLY, HUMAN, JASPAR, MALARIA, MOUSE, TFBSshape, WORM, and YEAST. The motif databases (updated on Decermber 8, 2015) were obtained from http://meme-suite.org/meme-software/Databases/motifs/motif_databases.12.11.tgz. We used FIMO from MEME 4.11.1 (Grant et al., 2011) with the arguments “*–bgfile TAIR10_500nt_trans_markov1 –max-stored-scores 10000000 –thresh 1e-4*” to identify genomewide hits for all MEME motifs; the search was done against the all-contigs derivative of *TAIR10_upstream_500_translation_start_20101028.fa*, in order to run FIMO against DNA sequences with standard nucleotide alphabets (ACGT). We tested three *p*-value thresholds for motif hits (10^-4^, 10^-5^, and 10^-6^) with three sets of genes for which motif prediction gave well-defined positive results, and for which we could thus tune FIMO thresholds empirically when trying to redetect an original gene set in a genomewide motif search: glucosinolate genes (which identified a MYB binding site), gene upregulated in *LGOoe* versus *LGOoe atml1-3* (which identified an HSF binding site), and known E2F target genes (which identified an E2F binding site). We found that, for all three of these controls, *p* ≤ 10^-5^ appeared to give the best balance between sensitivity and specificity in rediscovering known positive genes. We thus selected this as our FIMO threshold in all genomewide motif searches. The overall results (**Supplementary File S14**) were summarized with custom Perl scripts.

The statistical significance of overlaps between gene sets was evaluated by the two-sided exact binomial test at the 0.99 confidence level (i.e., with a 99% confidence interval), as implemented in the *stats* package of R (*binom.test*; R version 2.15.1; 2012-06-22).

## Availability of Data and Material

Seeds are available from the *Arabidopsis* Biological Resource Center (ABRC) with the following accession numbers: *atml1-3*, CS68906; *lgo-2*, CS69160; *ATML1p::LGO*, CS69161; and *ATML1p::LGO atml1-3*, CS69162.

Sequence files for quality-filtered, barcode-sorted, and barcode-trimmed RNA-seq reads have been deposited in the NCBI Sequence Read Archive (SRA^11^) under the accession numbers SRR3179593 (Col_WT, replicate 1), SRR3179623 (Col_WT, replicate 2), SRR3179642 (Col_WT, replicate 3), SRR3179988 (*atml1-3*, replicate 1), SRR3180064 (*atml1-3*, replicate 2), SRR3180073 (*atml1-3*, replicate 3), SRR3180080 (*lgo-2*, replicate 1), SRR3180088 (*lgo-2*, replicate 2), SRR3180318 (*lgo-2*, replicate 3), SRR3180447 (*LGOoe* [*ATML1p::LGO*], replicate 1), SRR3180481 (*LGOoe* [*ATML1p::LGO*], replicate 2), SRR3180517 (*LGOoe* [*ATML1p::LGO*], replicate 3), SRR3180541 (*LGOoe* [*ATML1p::LGO*] *atml1-3*, replicate 1), SRR3180542 (*LGOoe* [*ATML1p::LGO*] *atml1-3*, replicate 2), and SRR3180543 (*LGOoe* [*ATML1p::LGO*] *atml1-3*, replicate 3).

## Author Contributions

ES: bioinformatic analysis, writing the manuscript. AR: design of the experiment, isolation of RNA and creation of sequencing libraries, writing the manuscript.

## Conflict of Interest Statement

The authors declare that the research was conducted in the absence of any commercial or financial relationships that could be construed as a potential conflict of interest.

## Footnotes

1 http://hannonlab.cshl.edu/fastx_toolkit/index.html

2 https://github.com/agordon/fastx_toolkit/releases/download/0.0.14/fastx_toolkit-0.0.14.tar.bz2

3 https://github.com/agordon/libgtextutils/releases/download/0.7/libgtextutils-0.7.tar.gz

4 http://www.ncbi.nlm.nih.gov/sra

5 https://cran.r-project.org/web/packages/pheatmap/index.html

6 https://stat.ethz.ch/R-manual/R-patched/library/stats/html/hclust.html

7 http://www.ncbi.nlm.nih.gov/geo/query/acc.cgi?acc$=$GSE40322

8 http://www.ncbi.nlm.nih.gov/geo/geo2r/?acc$=$GSE40322

9 https://www.arabidopsis.org/download_files/Sequences/TAIR10_blastsets/TAIR10_cds_20101214_updated

10 http://bioinfo.cau.edu.cn/agriGO

11 http://www.ncbi.nlm.nih.gov/sra

## Abbreviations

ATML1: Arabidopsis thaliana MERISTEM LAYER1, AT4G21750
CDK: cyclin-dependent kinase
CKI: cyclin-dependent kinase inhibitor
CPR5: CONSTITUTUVE EXPRESSOR OF PATHOGENESIS RELATED GENES5 AT5G64930
GO: Gene ontology
HD-ZIP IV: homeodomain leucine zipper class IV
KRP: KIP-RELATED PROTEIN
LGO: LOSS OF GIANT CELLS FROM ORGANS, AT3G10525
LGOoe: overexpression of LGO throughout the epidermis (ATML1p::LGO)
PR: pathogenesis related
SIM: SIAMESE (AT5G04470)
SMR1: SIAMESE RELATED1, also known as LGO (AT3G10525)
TPM: transcripts per million
