# Supplementary material for: Transcriptomic Effects of the Cell Cycle Regulator LGO in Arabidopsis Sepals
Source: Front Plant Sci. 2016 Nov 22;7:1744. doi: 10.3389/fpls.2016.01744 (PMC5118908; doi:10.3389/fpls.2016.01744)
Supplement: Supplementary file 1 [file Data_Sheet_1.ZIP › Schwarz_Roeder_SupplementaryFiles_2016.09.26/Schwarz_Roeder_Supplementary_Material_2016.09.26.pdf]

Supplementary Material

The Cell Cycle Regulator LGO Exerts Transcriptomic Effects Primarily on Defense Response Genes in *Arabidopsis* Sepals

Erich M. Schwarz, Adrienne H. K. Roeder

*** Correspondence:** Adrienne Roeder: ahr75@cornell.edu

1 Supplementary Data

Supplementary File S01: Gene annotations. See the Excel file *SchwarzRoeder_2016.09.26_Supplementary_File_S01.xlsx*. This enumerates all protein-coding and control genes analyzed by RNA-seq, with identification numbers, aliases, descriptions, motifs, RNA-seq expression data, and instances of significantly changed gene expression between genotypes or batches. Its data columns are as follows:

Gene: generally, a given protein-coding gene in the TAIR10 release of the *Arabidopsis* genome database. All further data columns are pertinent to that particular gene; in particular, RNA-seq expression values for each gene were computed with RSEM. In addition to protein-coding genes from *Arabidopsis*, a small number of additional genes and sequences (rRNA genes, sequencing adapters, and GFP) were also included in this gene set, and had their RNA-seq expression values computed as negative controls for contamination (in the cases of rRNA and adapters) or for background noise (for GFP, given that it was known to be a true negative in all expression sets). Gene names follow standard genome-based identifications (e.g., "AT3G10525" rather than "LGO").

Aliases: human-readable names for genes, taken from the literature and archived in TAIR10 (e.g., "LGO" rather than "AT3G10525").

Short_desc: short descriptions of gene function, annotated in TAIR10.

Summary: summaries of gene function, annotated in TAIR10.

Comp_desc: automatic "computational" descriptions of gene function, annotated in TAIR10.

Domains: protein domains from InterPro encoded by a gene's product(s), with their accession numbers in brackets, annotated in TAIR10.

TF: annotations in this column denote whether a gene is predicted to encode a transcription factor, as defined in either of two databases for *Arabidopsis* (AtTFDB and PlantTFDB); the particular subtype of transcription factor is noted in brackets. For instance, ATML1 is annotated with "TF[HD-ZIP; Homeobox]".

GO_terms_genotype_primary: GO terms that both functionally annotate a given gene are most significantly with associated groups of genes changing their expression upon a change of genotype rather than upon a change of batch. In cases where the gene itself also significantly changes its

expression in a change of genotype or batch, the change is noted; in cases where the gene itself does not change expression, but the GO term is associated with some other gene that does significantly change, the term is noted with "not sig. gene".

GO_terms_batch_primary: GO terms that both functionally annotate a given gene and are most significantly with associated groups of genes changing their expression upon change of batch rather than upon a change of genotype, yet are also associated with a genotypic change. In cases where the gene itself also significantly changes its expression in a change of genotype or batch, the change is noted; in cases where the gene itself does not change expression, but the GO term is associated with some other gene that does significantly change, the term is noted with "not sig. gene".

GO_terms_batch_only: GO terms that both functionally annotate a given gene and are most significantly associated with a change of batch only, without any significant associations with a change of genotype. In cases where the gene itself also significantly changes its expression in a change of batch, the change is noted; in cases where the gene itself does not change expression, but the GO term is associated with some other gene that does significantly change, the term is noted with "not sig. gene".

GO_terms_other: GO terms that functionally annotate a given gene, but that are not associated significantly with groups of genes changing their expression given changes of either genotype or batch.

Noteworthy: annotations in this column denote specific genes that have been identified through past work as being required for normal development of giant cells and small cells in *Arabidopsis* sepals, or as containing a giant- or small-cell enhancer (Qu et al., 2014; Roeder et al., 2010; 2012).

log2FoldChange[*condition*]: For a given gene, this denotes the fold change of gene activity, computed by DESeq2 for significant differences between the following conditions (genotypes or batches): Col_WT vs. *lgo-2*; Col_WT vs. *atml1-3*; *atml1-3* vs. *lgo-2*; *LGOoe* vs. *lgo-2*; *LGOoe* vs. *atml1-3*; *LGOoe* vs. Col_WT; *LGOoe atml1-3* vs. *lgo-2*; *LGOoe atml1-3* vs. *atml1-3*; *LGOoe atml1-3* vs. Col_WT; *LGOoe* vs. *LGOoe atml1-3*; batch two vs. batch one; batch three vs. batch one, and batch three vs. batch two. Note that we have not included log2FoldChange values for changes in gene activity that DESeq2 failed to classify as significant (with an adjusted p-value threshold of ≤ 0.1). DESeq2 normalized read counts between the three batches for each condition before computing an overall log2FoldChange between two conditions. In each case, a positive value indicates that the first condition has stronger expression than the second condition.

padj[condition]: For a given gene, this denotes the p-value (adjusted for multiple hypothesis testing, via the Benjamini-Hochberg correction; hence, abbreviated as "padj") for any significant change of gene activity between two conditions, as listed above for "log2FoldChange[*condition*]". Also, as above, only padj values meeting the significance threshold of ≤ 0.1 are listed.

[*RNA-seq read set*]_TPM: for the gene in question, and for a given RNA-seq data set, this denotes the gene's activity as measured in that RNA-seq data set and computed by RSEM in Transcripts Per Million (TPM). RNA-seq data sets were generated for the genotypes Col_WT, *atml1-3*, *lgo-2*, *LGOoe*, and *LGOoe atml1-3*; for each genotype, there were three batch replicates (denoted by the suffixes '_rep1' through '_rep3'). All RNA-seq data from a given batch were generated from plants grown in parallel, so that environmental perturbations affecting sepal expression independently of

genotype would be discernable as common gene changes affecting all genes being compared between two batches.

[*RNA-seq read set*]_minTPM: for the gene in question, and for a given RNA-seq data set, this denotes the minimum estimate of that gene's activity as measured in that RNA-seq data set and computed by RSEM with a 99% confidence interval in Transcripts Per Million (minTPM). All genotypes and batch suffixes are as with "[*RNA-seq read set*]_TPM" above. Generating a statistically robust minimum estimate of gene activity allowed background noise to be distinguished from authentic but low expression levels above background, since the minTPM for a given gene could then be compared with the nominal TPM expression level for a GFP transgene (known to be a true negative) in that same data set.

[*RNA-seq read set*]_reads: for the gene in question, and for a given RNA-seq data set, this denotes a posterior mean estimate of the number of RNA-seq reads mapping to that gene as computed by RSEM, and with decimal fractions rounded off. All genotypes and batch suffixes are as with "**[*RNA-seq read set*]_TPM**" above. These read data were used to determine statistically significant changes of gene activity between conditions, via DESeq2.

Supplementary File S02: Differentially expressed genes. See the Excel file *SchwarzRoeder_2016.09.26_Supplementary_File_S02.xlsx*. These data have been excerpted from Supplementary File S01, for ease of study, and use a subset of the same data fields. Each sheet in the Excel file shows a different genotype or batch comparison, and lists all the significantly differentially expressed genes computed by DESeq2 for that comparison.

Supplementary File S03: edgeR gene significance scores. See the Excel file *SchwarzRoeder_2016.09.26_Supplementary_File_S03.xlsx*. On the first spreadsheet, a summary comparing DESeq2 vs. edgeR results is given, with the following data fields. **Comparison**: this denotes a given change between two conditions (genotype or batch, listed as "B vs. A") and each direction of change (increased or decreased gene expression in B, with respect to A) for which the number of genes with significantly changed expression was computed with either DESeq2 or edgeR. **DESeq2_genes**: this gives, for each comparison, the number of genes scored by DESeq2 as having had significantly changed expression. **edgeR_genes**: this gives, for each comparison, the number of genes scored by edgeR as having had significantly changed expression. **Overlap_genes**: this gives, for each comparison, the number of genes that were scored by both DESeq2 and edgeR as having had significantly changed expression. **Overlap_ratio**: this gives the ratio of overlap genes to edgeR genes (where this would involve division by zero, the value "n/a" is given). Notably, for most cases of there being one or more edgeR genes, the overlap ratios are quite high (between 0.875 and 1.000). In other words, most genes predicted by edgeR are a subset of genes predicted by DESeq2. **p_value**: the statistical significance of the observed overlap between DESeq2 and edgeR genes, versus the alternative of their having overlapped merely by chance (in a background of 27,416 protein-coding genes in TAIR10); all p-values were computed using two-sided exact binomial tests with a 99% confidence interval (Methods). On the second spreadsheet, all of the genes scored by edgeR as having had significantly changed expression are listed, with the following data fields. **log2FoldChange[*condition*]:** For a given gene, this denotes the fold change of gene activity, computed by edgeR for the same significant differences as in Supplementary File S01. Also as in

Supplementary File S01, log2FoldChange values are only given for genes classified as significant with an adjusted p-value threshold of ≤0.1, and read counts were normalized between all genotypes or batches for each condition. **padj[condition]:** For a given gene, this denotes the p-value (adjusted for multiple hypothesis testing; hence, abbreviated as "padj") for any significant change of gene activity between two conditions, as listed above for "log2FoldChange[*condition*]". Also, as above, only padj values meeting the significance threshold of ≤ 0.1 are listed.

Supplementary File S04: R^2 comparisons of gene activity levels between biological replicates. See the Excel file *SchwarzRoeder_2016.09.26_Supplementary_File_S04.xlsx*. This file contains the R^2 coefficient of determination comparing the TPM expression level of all genes in one replicate agains the TPM expression level of all genes in another replicate for the same genotype. An R^2 value of 1 would mean the two replicates had identical expression values for all genes. The column “replicate comparison" lists the two replicates are being compared. The column “R squared values” lists the R^2 value for that replicate comparison.

Supplementary File S05: Gene Ontology (GO) terms. See the Excel file *SchwarzRoeder_2016.09.26_Supplementary_File_S05.xlsx*. It contains the following data: GO terms for which the most significant effect (as measured by p-value) came from a genotypic comparison rather than a batch comparison; GO terms for which the most significant effect came from a batch comparison, but which did have significant p-values for genotypic comparisons as well; and GO terms that only had significant p-values for batch comparisons. These data are also listed by gene (in the columns "GO_term_genotype_primary", "GO_term_batch_primary", and "GO_terms_batch_only") in Supplementary File S01.

Supplementary File S06: Extended data for functions upregulated in sepals by *LGOoe* despite the absence of ATML1 (summarized in Table 3). See the Excel file *SchwarzRoeder_2016.09.26_Supplementary_File_S06.xlsx*. These terms are a subset of all the GO terms that are most significantly associated with changes in genotype, rather than changes in batch (Supplementary File S05, "GO terms-genotype primary" data sheet). The GO terms in this subset were selected for the following traits: they are significantly associated with genes that themselves have significantly higher expression in *LGOoe atml1-3* sepals than in *atml1-3* sepals (i.e., associated with genes that are upregulated by *LGOoe* despite the absence of ATML1); they are not significantly associated with genes that themselves have significant differences of expression in *LGOoe* versus *LGOoe atml1-3* sepals (i.e., not associated with genes that are driven by *LGOoe* in an ATML1-dependent manner); and they are significantly associated with at least one other set of genes that themselves exhibited significantly changed expression in at least one genotypic change involving *LGOoe*, other than *LGOoe atml1-3* versus *atml1-3* (i.e., their being associated with *LGOoe*-based genotypic changes is not an isolated result). GO term describes functions significantly overrepresented among genes that were more strongly expressed in *LGOoe atml1-3* sepals than in *atml1-3* sepals. p-value [condition] lists, for a given GO term, the p-values for all sets of genes that exhibited significant changes of expression under genotypic and batch changes and that were significantly associated with that GO term. In some, but not all cases, the most significant p-value

was associated with genes more strongly expressed in *LGOoe atml1-3* versus *atml1-3*. All GO annots. gives the total number of genes in the genome annotated for a given GO term, divided by the total number of genes in the genome annotated for any GO term; the decimal fraction of this ratio is given in brackets. Cond. GO annots. gives the total number of genes in the genome that both are annotated for a given GO term and exhibit significant changes of gene expression under a conditional change associated with that same GO term, divided by the total number of genes in the genome exhibiting such changes of gene expression; the decimal fraction of this ratio is given in brackets. For GO terms with strong p-values, it is easy to compare the ratios in All GO annots. and Cond. GO annots. visually and see the enrichment of the GO term among genes with conditionally changed expression. Gene count is given for the number of genes that are associated both with the GO term and with one of its associated genotypic changes; Genes provides their individual identities.

Supplementary File S07: Extended data for functions expressed in *LGOoe* sepals in an *ATML1*-dependent manner (summarized in Table 4). See the Excel file *SchwarzRoeder_2016.09.26_Supplementary_File_S07.xlsx*. These terms are a subset of all the GO terms that are most significantly associated with changes in genotype, rather than changes in batch (Supplementary File S05, "GO terms-genotype primary" data sheet). The GO terms in this subset were selected for the following traits: they are significantly associated with genes that themselves have significantly higher expression in *LGOoe* sepals than in *LGOoe atml1-3* sepals (i.e., associated with genes that are expressed in *LGOoe* sepals in an ATML1-dependent manner); and they are significantly associated with at least one other set of genes that themselves exhibited significantly changed expression in at least one genotypic change involving *LGOoe*, other than *LGOoe* versus *LGOoe atml1-3* (i.e., their being associated with *LGOoe*-based genotypic changes is not an isolated result). The data fields GO term, p-value [condition], All GO annots., Cond. GO annots., Gene count, and Genes are as in Supplementary File S06.

Supplementary File S08: Extended data for functions more expressed in *LGOoe* sepals than in either *lgo-2* or *atml1-3* sepals (summarized in Table 5). See the Excel file *SchwarzRoeder_2016.09.26_Supplementary_File_S08.xlsx*. These terms are a subset of all the GO terms that are most significantly associated with changes in genotype, rather than changes in batch (Supplementary File S05, "GO terms-genotype primary" data sheet). The GO terms in this subset were selected for the following traits: they are significantly associated with genes that themselves have significantly higher expression in *LGOoe* sepals than in *lgo-2* sepals, and that also have significantly higher expression in *LGOoe* sepals than in *atml1-3* sepals; they are not significantly associated with genes that themselves have significant differences of expression either in *LGOoe atml1-3* sepals versus in *atml1-3* sepals, or in *LGOoe* sepals versus *LGOoe atml1-3* sepals (i.e., they are not associated either with genes that are upregulated by *LGOoe* despite the absence of ATML1, or with genes that are expressed in *LGOoe* sepals in an ATML1-dependent manner); and (by definition) they are associated with changed gene expression in at least two different genotypic changes involving *LGOoe* (i.e., their being associated with *LGOoe*-based genotypic changes is not an isolated result). The data fields GO term, p-value [condition], All GO annots., Cond. GO annots., Gene count, and Genes are as in Supplementary File S06.

Supplementary File S09: Extended data for functions downregulated in sepals by *LGOoe* despite the absence of ATML1. See the Excel file *SchwarzRoeder_2016.09.26_Supplementary_File_S09.xlsx*. These terms are a subset of all the GO terms that are most significantly associated with changes in genotype, rather than changes in batch (Supplementary File S05, "GO terms-genotype primary" data sheet). The GO terms in this subset were selected for the following traits: they are significantly associated with genes that themselves have significantly lower expression in *LGOoe atml1-3* sepals than in *atml1-3* sepals (i.e., associated with genes that are downregulated by *LGOoe* despite the absence of ATML1); they are not significantly associated with genes that themselves have significant differences of expression in *LGOoe* versus *LGOoe atml1-3* sepals (i.e., not associated with genes that are driven by *LGOoe* in an ATML1-dependent manner); and they are significantly associated with at least one other set of genes that themselves exhibited significantly changed expression in at least one genotypic change involving *LGOoe*, other than *LGOoe atml1-3* versus *atml1-3* (i.e., their being associated with *LGOoe*-based genotypic changes is not an isolated result). The data fields GO term, p-value [condition], All GO annots., Cond. GO annots., Gene count, and Genes are as in Supplementary File S06.

Supplementary File S10: Extended data for functions repressed in *LGOoe* sepals in an *ATML1*-dependent manner. See the Excel file *SchwarzRoeder_2016.09.26_Supplementary_File_S10.xlsx*. These terms are a subset of all the GO terms that are most significantly associated with changes in genotype, rather than changes in batch (Supplementary File S05, "GO terms-genotype primary" data sheet). The GO terms in this subset were selected for the following traits: they are significantly associated with genes that themselves have significantly lower expression in *LGOoe* sepals than in *LGOoe atml1-3* sepals (i.e., associated with genes that are repressed in *LGOoe* sepals in an ATML1-dependent manner); and they are significantly associated with at least one other set of genes that themselves exhibited significantly changed expression in at least one genotypic change involving *LGOoe*, other than *LGOoe* versus *LGOoe atml1-3* (i.e., their being associated with *LGOoe*-based genotypic changes is not an isolated result). The data fields GO term, p-value [condition], All GO annots., Cond. GO annots., Gene count, and Genes are as in Supplementary File S06.

Supplementary File S11: Hierarchical GO graph for genes significantly upregulated in *LGOoe atml1-3* compared to *atml1-3*. See the PDF file *SchwarzRoeder_2016.09.26_Supplementary_File_S11.pdf*. Hierarchical graph made with AgriGO, showing the relationship of the significant GO terms associated the genes upregulated in *LGOoe atml1-3* versus *atml1-3*. The significance of the GO term is represented as a heat map with highly significant adjusted p-values ($<10^{-10}$) in red and terms just meeting the significance cutoff of adjusted p-values (<0.05) in yellow. Each box contains the GO term, the adjusted p-value, and the number of genes associated with the GO term over (/) the genes upregulated in *LGOoe atml1-3* versus *atml1-3*, versus (|) the number of genes associated with the GO term in the genome over (/) the total number of genes in the genome. All values were calculated in AgriGO; these may differ slightly from the values calculated with FUNC.

Supplementary File S12: Hierarchical GO graph for genes significantly upregulated in *LGOoe* compared to *LGOoe atml1-3*. See the PDF file *SchwarzRoeder_2016.09.26_Supplementary_File_S12.pdf*. Hierarchical graph made with AgriGO, showing the relationship of the significant GO terms associated the genes upregulated in *LGOoe versus LGOoe atml1-3*. The significance of the GO term is represented as a heat map with highly significant adjusted p-values ($<10^{-10}$) in red and terms just meeting the significance cutoff of adjusted p-values (<0.05) in yellow. Each box contains the GO term, the adjusted p-value, and the number of genes associated with the GO term over (/) the genes upregulated in *LGOoe* versus *LGOoe atml1-3* versus (|) the number of genes associated with the GO term in the genome over (/) the total number of genes in the genome. All values were calculated in AgriGO; these may differ slightly from the values calculated with FUNC.

Supplementary File S13: Hierarchical GO graph for genes significantly upregulated in *LGOoe* compared to *lgo-2*. See the PDF file *SchwarzRoeder_2016.09.26_Supplementary_File_S13.pdf*. Hierarchical graph made with AgriGO, showing the relationship of the significant GO terms associated the genes upregulated in *LGOoe* versus *lgo-2*. The significance of the GO term is represented as a heat map with highly significant adjusted p-values ($<10^{-10}$) in red and terms just meeting the significance cutoff of adjusted p-values (<0.05) in yellow. Each box contains the GO term, the adjusted p-value, and the number of genes associated with the GO term over (/) the genes upregulated in *LGOoe* versus *lgo-2* versus (|) the number of genes associated with the GO term in the genome over (/) the total number of genes in the genome. All values were calculated in AgriGO; these may differ slightly from the values calculated with FUNC.

Supplementary File S14: Summary of non-coding DNA motifs. See the Excel file *SchwarzRoeder_2016.09.26_Supplementary_File_S14.xlsx*. This summarizes the results of searches for non-coding DNA motifs (of potential cis-regulatory function) with MEME, carried out against 500-nt 5' flanks from various sets of genes, and the subsequent results of using these motifs in FIMO searches to rescan the 500-nt 5'-flanks for all 27,416 protein-coding genes in *Arabidopsis*. If a given input gene set (e.g., genes annotated for the GO term *glucosinolate biosynthetic process* [GO:0019761]) happened to be highly enriched for a non-coding motif, such a motif was more likely to be discovered, and had some possibility of being relevant to that gene set. Rescanning the entire genome then allowed us to test the possibility of relevance, in two ways. First, it tested whether the motif could rediscover most or all of the genes in its original gene group, even when scanning the entire genome. Second, it also tested whether that motif only achieved rediscovery of its original MEME query gene set by being highly promiscuous (i.e., by "discovering" many thousands of other genes), or whether it indeed had hits only to a small fraction of all protein-coding genes in the genome.

The data columns of this table are as follows:

Data: the name of the gene list used to seed the MEME search. Names generally match GO terms, gene expression categories, previously published gene sets, or their intersection in one case (the *cpr5* gene set intersected with the full 1,341-gene differentially expressed sepal set).

Motif: an integer denoting the "motif name" given by MEME for a given predicted motif. These nondescript names are only useful when coupled with data names.

Consensus: the consensus sequence of the motif as computed by MEME.

Rev_comp_consensus: the reverse-complement of MEME's computed consensus. In some cases, on comparison of motifs to previously published motifs, the reverse-complement rather than the original consensus aligned with its predecessor. In these cases, we have selected the reverse-complement to name the motif in the main text.

Orig_search_genes: the number of Arabidopsis genes in the original MEME search set.

Orig_positive_genes: out of the genes in **Orig_search_genes**, the number that yielded initial positive hits for a newly predicted motif.

Redisc_positive_genes: out of the genes in **Orig_search_genes**, the number that were successfully redetected by that motif in a genomewide search with FIMO, at a threshold of $p \leq 1 \cdot 10^{-05}$.

All_genes: the total number of genes searched in the genomewide FIMO rescan with a motif; always 27,416, but placed in the table for ease of visual comparison.

All_sepal_genes: the total number of genes with some significant genotypic change of expression in sepals; always 1,341, but placed in the table for ease of visual comparison.

Genomewide_hits: for a given motif, how many genes in the entire genome it detected as hits when used for a FIMO search.

Sepalwide_hits: for a given motif with **Genomewide_hits**, the subset of those genes that also fell within the sepal-expressed 1,341-gene set.

Redisc_orig_query_ratio: for a given motif, the ratio between the number of genes in its query set that it rediscovered in a genomewide FIMO search to the number of genes in that original query set.

Redisc_orig_pos_ratio: for a given motif, the ratio between the number of originally positive genes in its query set that it rediscovered in a genomewide FIMO search to the number of originally positive genes in that original query set. Note that this is not identical to Redisc_orig_query_ratio, because an initial MEME search can find a motif in a subset of its original query gene set. Conceivably, such a low-frequency initial motif might well be highly specific, but not for the entire large query gene set in which it was discovered. Checking the Redisc_orig_pos_ratio value affords the possibility to detecting such motifs.

Gwide_hit_ratio: the ratio of Genomewide_hits to All_genes. The larger this ratio, the more likely a given motif is to be utterly generic (if it is real at all).

Sepalwide_hit_ratio: the ratio of Sepalwide_hits to All_sepal_genes.

Query_enrichment: the ratio of Redisc_orig_query_ratio to Gwide_hit_ratio. The higher this is, the less likely that redetection of the original query genes is due to a chance genomewide discovery rate, and the more likely it is to reflect genuine specificity of the motif to its query gene set.

Sepal_enrichment: the ratio of Sepalwide_hit_ratio to Gwide_hit_ratio. The higher this is, the less likely that redetection of the original query genes is due to a chance genomewide discovery rate, and

the more likely it is to reflect genuine specificity of the motif to all genes differentially expressed in the sepal.

Query_p-value: the probability that Query_enrichment was due to chance, as computed with the two-sided binomial test and a 99% confidence interval.

Sepal_p-value: the probability that Sepal_enrichment was due to chance, as computed with the two-sided binomial test and a 99% confidence interval.

Supplementary File S15: Functions associated with previously published gene groups of interest. See the Excel file *SchwarzRoeder_2016.09.26_Supplementary_File_S15.xlsx*. These groups include the following: genes upregulated in *cpr5* versus *cpr5 sim lgo* plants (Wang et al., 2014); functions encoded by E2F target genes in *Arabidopsis* (Vandepoele et al., 2005); functions encoded by trichome-specific genes, as defined by Jakoby et al. (Jakoby et al., 2008); and functions encoded by trichome-specific genes, as defined by Marks et al. (Marks et al., 2009). In cases where a given GO term overlaps with the GO terms in Supplementary Files S06-S08, it is marked in blue. There are 24 such cases for the *cpr5* GO terms, none for the E2F GO terms, two for the Jakoby trichome GO terms, and three for the Marks trichome GO terms.

Supplementary File S16: Custom Perl scripts used for bioinformatics. See the text file *SchwarzRoeder_2016.09.26_Supplementary_File_S16.txt*.

Supplementary File S17: Adapter sequences provided to Trimmomatic. See the text file *SchwarzRoeder_2016.09.26_Supplementary_File_S17.txt*.

Supplementary File S18: Barcodes provided to *fastx_barcode_splitter.pl*. See the text file *SchwarzRoeder_2016.09.26_Supplementary_File_S18.txt*.

Supplementary File S19: RNA-seq libraries. See the Excel file *SchwarzRoeder_2016.09.26_Supplementary_File_S19.xlsx*. It contains the following data for all RNA-seq libraries: mean insert sizes and standard deviations; numbers of quality-filtered reads mapped to the *Arabidopsis* transcriptome by RSEM.

Supplementary File S20: DNA sequence information. This lists identities and sources for all DNA sequences used in the *Arabidopsis* RSEM gene index. See the Word document file *SchwarzRoeder_2016.09.26_Supplementary_File_S20.docx*.

Supplementary File S21: Genes expressed. See the Excel file *SchwarzRoeder_2016.09.26_Supplementary_File_S21.xlsx*. It contains the following data spreadsheets. Overall expr.: numbers of genes expressed per genotype, and overall number of genes with detectable expression. Note that we counted a gene as expressed above background noise levels in a given RNA-seq replicate if its minTPM (minimum TPM expression value in a 99% credibility interval) was greater than the nominal expression level (measured in TPM, and in that replicate) computed by RSEM for a known negative gene, GFP (see Methods). Replicate expr.: for each RNA-seq replicate, the following data are summarized. Number of *Arabidopsis* protein-coding genes expressed above background; the maximum observed expression level; the minimum observed expression level; the maximum and minimum observed minTPM levels (i.e., minimum estimated expression, with a 99% credibility interval); and the nominal expression level of GFP. All expression values are in TPM. Sig. cond. genes: for each change between two conditions (batch or genotype) and each direction of change (increased or decreased gene expression), the number of genes changing with statistical significance (as evaluated by DESeq2; see Methods) is listed.

Supplementary File S22: DESeq2 commands. Specific R commands used to generate DESeq2 significances (adjusted p-values) for comparisons of gene activity between genotypes or batches. See the text file *SchwarzRoeder_2016.09.26_Supplementary_File_S22.txt*.

Supplementary File S23: edgeR commands. Specific R commands used to generate edgeR significances (adjusted p-values) for comparisons of gene activity between genotypes or batches. See the text file *SchwarzRoeder_2016.09.26_Supplementary_File_S23.txt*.

Supplementary File S24: Previously published sets of genes. See the Excel file *SchwarzRoeder_2016.09.26_Supplementary_File_S24.xlsx*. It contains the following gene lists extracted from previously published work: genes upregulated in *cpr5* versus *cpr5 sim lgo* plants (Wang et al., 2014); E2F target genes in *Arabidopsis* (Vandepoele et al., 2005); trichome-specific genes, as defined by Jakoby et al. (Jakoby et al., 2008); trichome-specific genes, as defined by Marks et al. (Marks et al., 2009); and genes bound by KRP5, as observed by Jégu et al. (Jegu et al., 2013).
