# Supplementary material for: Transcriptomic Effects of the Cell Cycle Regulator LGO in Arabidopsis Sepals
Source: Front Plant Sci. 2016 Nov 22;7:1744. doi: 10.3389/fpls.2016.01744 (PMC5118908; doi:10.3389/fpls.2016.01744)
Supplement: Supplementary file 1 [file Data_Sheet_1.ZIP › Schwarz_Roeder_SupplementaryFiles_2016.09.26/SchwarzRoeder_2016.09.26_Supplementary_File_S20.docx]

**Supplementary File S20:** Identities and sources for all DNA sequences used in the *Arabidopsis* RSEM gene index.

**1. *Arabidopsis* coding sequences from TAIR10**

**Source:** *https:/​/​www.arabidopsis.org/​download_​files/​Sequences/​TAIR10_​blastsets/​TAIR10_​cds_​20101214_​updated*

**Description:** This sequence file "contains all TAIR10 CDS sequences ... updated on April 16, 2012 to remove a handful of incorrect gene symbols and full names". Documentation is in: *https:/​/​www.arabidopsis.org/​download_​files/​Sequences/​TAIR10_​blastsets/​Readme_​blastdatasets_​TAIR10.txt*

**2. *Arabidopsis* rRNA coding sequences from TAIR10**

**Source:** the identities of these DNA sequences were first established by searching TAIR10, but the actual DNA sequences were downloaded from GenBank, with the following accession numbers. Note that only subsets of the full DNA records, corresponding to annotated coding sequences, were extracted and used for the RSEM gene index.

Athal_25S_RRNA.t1 -- nt 848-4222 from gi|16131|emb|X52320.1| *Arabidopsis thaliana* genes for 5.8S rRNA and 25S rRNA with 18S rRNA fragment

Athal_18S_RRNA.t1 -- nt 88-1891 from gi|16506|emb|X16077.1| *Arabidopsis thaliana* 18S rRNA gene

Athal_5.8S_RRNA.t1 -- nt 497-660 from gi|16131|emb|X52320.1| *Arabidopsis thaliana* genes for 5.8S rRNA and 25S rRNA with 18S rRNA fragment

**Description:** including these as genes provided a negative control, testing for any rRNA contaminants that passed through the experimental protocol into RNA-seq reads.

**3. Sepal adaptor sequences**

Source: These were identical to the sequences in Additional File X2.

**Description:** including this as a nominal gene in the RNA-seq analysis provided a negative control, testing for any adaptor-contaminated reads that failed to be filtered with Trimmomatic.

**4. GFP coding sequence (*Arabidopsis*-optimized)**

**Source:** the DNA sequence was downloaded from GenBank, as follows. Note that only a subset of the full DNA record, corresponding to annotated protein-coding sequence, was extracted and used for the RSEM gene index.

GFP_coding_seq.t1 -- nt 21-812 from gb|U87625.1|SCU87625 Synthetic construct modified green fluorescent protein GFP-ER (mfgp4-ER) mRNA, complete cds

**Description:** this is a sequence used for transgenic constructs in the Roeder laboratory. Since none of the tissues in this study had GFP transgenes, the computed level of gene expression for this transgene (in TPM) provided a convenient, empirical measurement for background noise in this RNA-seq analysis.
