# Supplementary figures and images for: Transcriptomic Effects of the Cell Cycle Regulator LGO in Arabidopsis Sepals
Source: Front Plant Sci. 2016 Nov 22;7:1744. doi: 10.3389/fpls.2016.01744 (PMC5118908; doi:10.3389/fpls.2016.01744)

***LGOoe atml1-3* vs. *atml1-3***

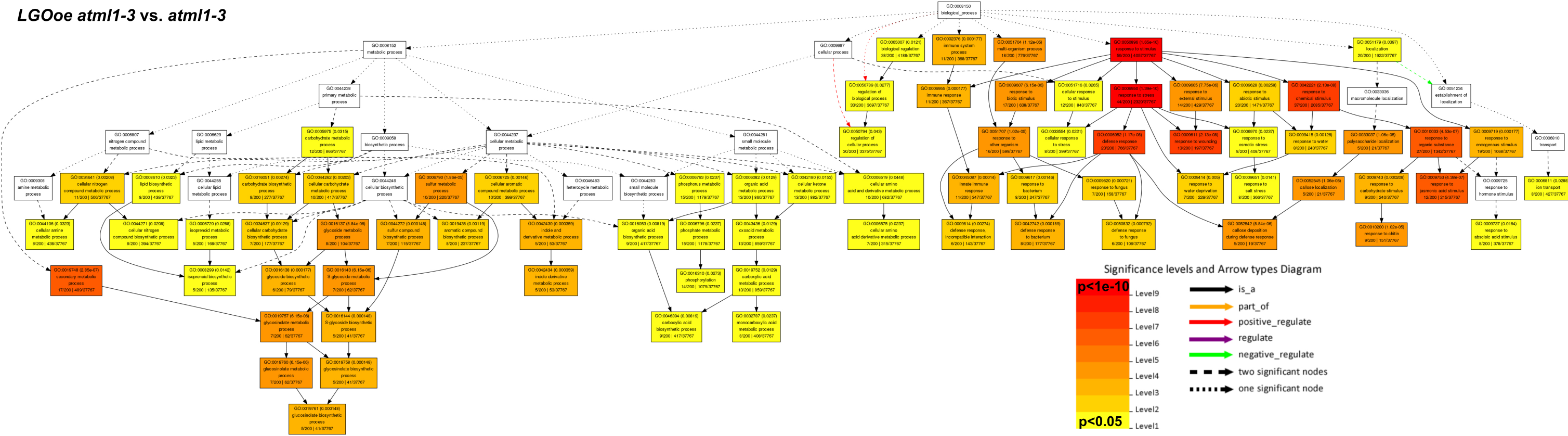

Supplement: Supplementary file 1 [file Data_Sheet_1.ZIP › Schwarz_Roeder_SupplementaryFiles_2016.09.26/SchwarzRoeder_2016.09.26_Supplementary_File_S11.pdf]

## LGOoe vs. lgo-2

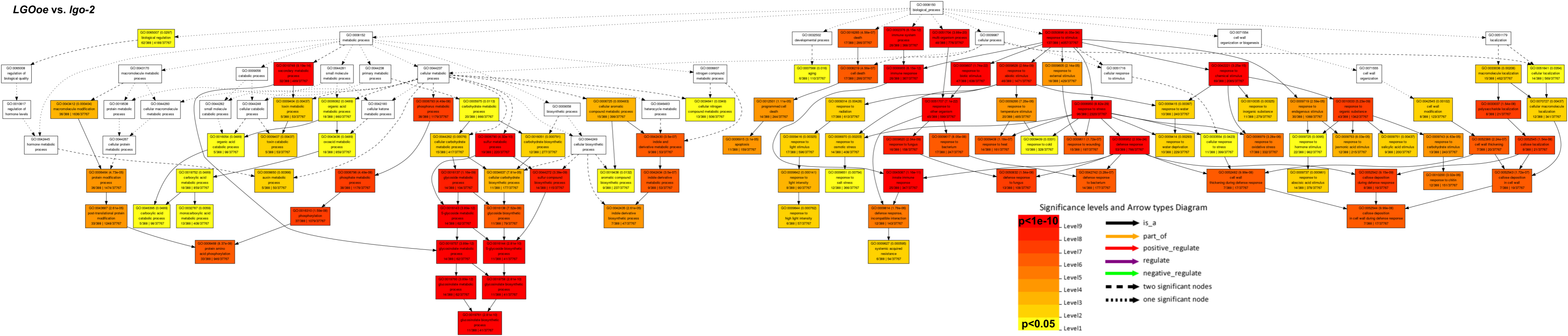

Supplement: Supplementary file 1 [file Data_Sheet_1.ZIP › Schwarz_Roeder_SupplementaryFiles_2016.09.26/SchwarzRoeder_2016.09.26_Supplementary_File_S13.pdf]
